# Overlap phenotypes of the left ventricular noncompaction and hypertrophic cardiomyopathy with complex arrhythmias and heart failure induced by the novel truncated DSC2 mutation

**DOI:** 10.1186/s13023-021-02112-9

**Published:** 2021-11-24

**Authors:** Yubi Lin, Jiana Huang, Zhiling Zhu, Zuoquan Zhang, Jianzhong Xian, Zhe Yang, Tingfeng Qin, Linxi Chen, Jingmin Huang, Yin Huang, Qiaoyun Wu, Zhenyu Hu, Xiufang Lin, Geyang Xu

**Affiliations:** 1 The Center of Cardiovascular Diseases, The Department of Cardiology, Radiology and Ultrasonography, Guangdong Provincial Key Laboratory of Biomedical Imaging, The Fifth Affiliated Hospital, Sun Yat-Sen University, Zhuhai, 519000 China; 2 Reproductive Center, The Six Affiliated Hospital, Sun Yat-Sen University, Guangzhou, 510000 China; 3 Department of Physiology, The School of Medicine of Jinan University, Guangzhou, 510000 China; 4 Department of Physiology, Yong Loo Lin School of Medicine, National University of Singapore, Singapore, 117593 Singapore

**Keywords:** Left ventricular noncompaction cardiomyopathy, Hypertrophic cardiomyopathy, Heart failure, desmocollin2, Desmosome

## Abstract

**Background:**

The left ventricular noncompaction cardiomyopathy (LVNC) is a rare subtype of cardiomyopathy associated with a high risk of heart failure (HF), thromboembolism, arrhythmia, and sudden cardiac death.

**Methods:**

The proband with overlap phenotypes of LVNC and hypertrophic cardiomyopathy (HCM) complicates atrial fibrillation (AF), ventricular tachycardia (VT), and HF due to the diffuse myocardial lesion, which were diagnosed by electrocardiogram, echocardiogram and cardiac magnetic resonance imaging. Peripheral blood was collected from the proband and his relatives. DNA was extracted from the peripheral blood of proband for high-throughput target capture sequencing. The Sanger sequence verified the variants. The protein was extracted from the skin of the proband and healthy volunteer. The expression difference of desmocollin2 was detected by Western blot.

**Results:**

The novel heterozygous truncated mutation (p.K47Rfs*2) of the *DSC2* gene encoding an important component of desmosomes was detected by targeted capture sequencing. The western blots showed that the expressing level of functional desmocollin2 protein (~ 94kd) was lower in the proband than that in the healthy volunteer, indicating that *DSC2* p.K47Rfs*2 obviously reduced the functional desmocollin2 protein expression in the proband.

**Conclusion:**

The heterozygous *DSC2* p.K47Rfs*2 remarkably and abnormally reduced the functional desmocollin2 expression, which may potentially induce the overlap phenotypes of LVNC and HCM, complicating AF, VT, and HF.

## Introduction

The left ventricular noncompaction cardiomyopathy (LVNC) is a rare subtype of cardiomyopathy (CM) and is characterized by predominant left ventricular trabeculations with deep intertrabecular recesses and thinning of the compact epicardium [1]. LVNC can coexist with dilated (DCM), hypertrophic (HCM), restrictive (RCM), and arrhythmogenic cardiomyopathy (ACM) [2–4]. LVNC is an inherited cardiac disease, classified as primary genetic CM, and associated with high risks of syncope, chest pain, heart failure (HF), malignant arrhythmia, sudden cardiac death (SCD), and thromboembolism [5]. A recent systematic review illustrated that the risks of thromboembolism and ventricular arrhythmias in LVNC are similar to DCM. Additionally, LVNC has a higher incidence of HF hospitalization than DCM. The low left ventricular ejection fraction (LVEF) induced by LVNC is associated with a poor prognosis [6]. A previous genetic study indicated that the mutations of genes encoding sarcomeric proteins account for up to 30% of LVNC [7]. Other common mutations of genes encoding cytoskeletal, Z-line, and mitochondrial proteins include myosin heavy chain (*MYH7*), protein-binding protein C myosin (*MYBPC3*), tropomyosin alfa (*TPM1*), myocardial actin (*ACTC1*), troponin T (*TNNT2*), and cardiac troponin I. The less common mutations of genes inducing LVNC include Z-band protein mutations, such as Cypher/ZASP cytoskeleton protein, alpha-distrobrevin (*DTNA*), calcium transport proteins, calsequestrin, phospholamban, membrane proteins (A/C lamina), para-zinc-finger transcription factor [8], and mitochondrial enzymes. Our study identifies a proband with rare overlap phenotypes of LVNC and HCM from a Chinese Han family and explores the potential pathogenesis via genetic screening and molecular experiments.

## Material and methods

### Patients and clinical variables

All participants signed informed consent. All procedures performed in the study involving human participants were in accordance with the Declaration of Helsinki and ethical standards and were approved by the Medicine Ethics Committee of the Fifth Affiliated Hospital of Sun Yat-sen University (No. 2020K216-1).

In accordance with previous studies [1, 9], LVNC was diagnosed by cardiologists based on clinical presentation and interpretation of echocardiogram and cardiac magnetic resonance imaging (CMRI) results by using the Jenni and Petersen criteria, respectively [10, 11]. The LVNC diagnosis was based on the presence of an end-systolic ratio of the noncompacted layer to compacted layer above 2 (NC/C ≥ 2.0) on echocardiography [10] and/or an end-diastolic ratio between the noncompacted and compacted layer greater than 2.3/2.0 (NC/C ≥ 2.3/2.0) in the long/short-axis view of CMRI with specificity and negative predictive values of 99% [11]. The left ventricular systolic dysfunction was defined as systolic LVEF < 50% by echocardiogram [1]. The clinical assessment also included physical examination and 12-lead and Holter electrocardiograms (ECGs). The N-terminal of B-type natriuretic peptide precursor (NT-proBNP) assays was performed at a central independent laboratory using a commercially available kit (Roche Diagnostics, Mannheim, Germany). We obtained the skin and subcutaneous tissue of the upper left limb of the patient (II: 1) and healthy volunteer through small surgery and carried out molecular biological detection. The healthy volunteer was a 37-year-old male without cardiac diseases verified by ECG and echocardiogram.

### CMRI

Imaging was acquired using the 1.5 T MR imaging system (MAGNETOM Aera, Siemens Healthcare, Germany) by using a 6-channel phased-array coil. After scout and reference scans, functional and geometric assessments were achieved using ECG-gated, cine steady-state-free precession images in standard long- (2-, 3-, and 4-chamber views) and short-axis orientations with full ventricle coverage from basis to apex. Left and right ventricular outflow tract cine sequences were also obtained. Cine images with a temporal resolution of approximately 40 ms were obtained. Additional pulse sequences, including T1WI and T2WI of blood-suppressed double inversion recovery fast spin-echo, were acquired in a standard location before contrast administration. Then, the dynamic enhancement was performed during the administration of 0.2 mmol/kg contrast agent (Gadovist, Bayer Healthcare, Berlin, Germany) with 50 frames. Late gadolinium-enhanced imaging was performed with 20 min delay and phase-sensitive inversion recovery sequence to detect myocardial scarring or fibrosis. CMRI analyses were performed using SigoVia (Siemens). The functional and geometric left ventricular indices, including left and right ventricular end-diastolic volumes, LVEF and right ventricular ejection fraction, indexed left ventricular compacted mass with papillary muscles, indexed left ventricular compacted and noncompacted mass, indexed left ventricular noncompacted mass, and their ratio was determined. In addition, the end-diastolic extent of compacted and noncompacted myocardium and their ratio were measured in one long axis geometry.

### Collection of human specimens and target capture sequencing

Skin biopsies were collected from the upper left arm of the proband and healthy volunteer. Protein was extracted from tissues. Peripheral blood was collected from the proband and his relatives. DNA was extracted (using D3537-02#MagPure Buffy Coat DNA Midi KF Kit, Magen, Beijing, China) from the peripheral blood of proband for high-throughput target capture sequencing using Gene fragment capture chip (MGI BGI EXOME V4, Shenzhen, China) and MGIseq-2000 sequencer (MGI, Shenzhen, China). The panel of common risk genes associated with CMs and arrhythmias (Table 1) was detected in the proband (II: 1). SNPs and Indels were annotated using a pipeline, in which all insertion and deletion variants occurring at coding regions were considered damaging. Nonsynonymous SNPs were predicted using the SIFT (http://sift.jcvi.org/www/), PolyPhen-2 (Polymorphism Phenotyping v2, http://genetics.bwh.harvard.edu/pph2/), and MetaSVM [12]. Variants were classified as “pathogenic (P)”, “likely pathogenic (LP)”, “uncertain significance (US or VUS)”, “likely benign (LB)”, or “benign (B)” by using the InterVar tool [13] following the 2015 ACMG/ACP guidelines [14]. Variants in predisposing genes associated with hereditary arrhythmias and CMs were screened, and the filtering criteria were as follows: (1) same variants in the WES data; (2) missense, nonsense, insertion, and deletion variants; and (3) SNPs with minor allele frequency < 0.01 according to the SNP database of National Center. Other familial members were validated using the Sanger sequencing for potentially pathogenic genes. Details are shown in our previous research [15].

**Table 1:** Susceptible genes of inherited cardiomyopathy and arrhythmia detected in the proband II: 1

*A2M, AARS2, ABCA1, ABCC6, ABCC9, ABCD4, ABCG5, ABCG8, ACE, ACTA2, ACTC1, ACTN2, ACVRL1, AGT, AGTR1, AKAP9, AKAP10, ALPK3, AMPD1, ANK2, ANK3, APBB2, APOA2, APOB, APP, ARFGEF2, ARSA, ASPA, BAG3, BCS1L, BLM, BLMH, BMP1, BMPR2, BRAF, CACNA1C, CACNA2D1, CACNB2, CALR3, CASQ2, CAV3, CBL, CBS, CCM2, COL1A1, COL1A2, COL3A1, COL4A1, COL4A2, COL5A1, COL5A2, COX10, COX15, CRELD1, CRTAP, CRYAB, CSRP3, CST3, CTNNA3, DES, DPP6, DSC2, DSG2, DSP, DTNA, ELN, EMD, ENG, ENPP1, ERCC6, ERCC8, ESR1, EYA4, F2, F5, F7, F12, F13A1, FBN1, FKBP10, FKBP12, FKTN, FLNC, FOXRED1, GALC, GATA4, GATA6, GATAD1, GCLC, GDF1, GFAP, GHR, GJA1, GJA5, GLA, GNAI2, GPD1L, GSN, GUCY1A3, HADHB, HBB, HCFC1, HCN4, HEY2, HFE, HTRA1, IFITM5, ITGB3, ITM2B, JAG1, JAK2, JARID2, JPH2, JUP, KCNA5, KCNE1, KCNE2, KCNE3, KCNH2, KCNJ2, KCNJ5, KCNMB1, KCNQ1, KRAS,TAZ, KRIT1, LAMA4, LAMP2, LDB3, LDLR, LDLRAP1, LMBRD1, LMNA, LMX1B, LRP6, LTA, MAPT, MED13L, MEF2A, MIB1, MIB2, MIPEP, MLC1, MLYCD, MMACHC, MMADHC, MPO, MTHFR, MTR, MTRR, MYBPC3, MYH3, MYH6, MYH7, MYH11, MYL2, MYL3, MYLK2, MYOT, MYOZ2, MYPN, NEXN, NDUFA2, NDUFA9, NDUFA10, NDUFA12, NDUFAF2, NDUFAF6, NDUFS3, NDUFS4, NDUFS7, NDUFS8, NEBL, NKX2.5, NKX2.6, NNT, NONO, NOS3, NOTCH1, NPPA, NRAS, NRG1, NSD1, OBSCN, PCSK9, PDCD10, PDE4D, PDLIM3, PKD1, PKD2, PKP2, PLAU, PLEKHM2, PLN, PLP1, PlXND1, PMP22, PPIB, PRDM16, PRKAG2, PRKAR1A, PSEN1, PSEN2, PTPN11, RAF1, RASA1, RBM20, REN, RNF213, RYR2, SORL1, RPS6KA3, SCN1B, SCN3B, SCN4B, SCN5A, SDHA, SDHAF1, SDHD, SERPINE1, SERPINF1, SERPINH1, SGCD, SH2B1, SHOC2, SLC6A4, SLC25A4, SLC39A8, SMAD3, SMAD4, SMC1A, SNTA1, SOS1, SP7, SPARC, STRA6, SURF1, TBX1, TBX5, TBX20, TCAP, TGFB2, TGFB3, TGFBR1, TGFBR2, TLL1, TMEM38B, TMEM43, TMEM70, TNNC1, TNNI2, TNNI3, TNNT2, TNNT3, TPM1, TPM2, TREX1, TRPM4, TSC2, TSPYL1, TTN, TTR, VCL, VKORC1, WNT1, YWHAE, ZFPM2*	

### ACMG classification

According to ACMG standards and guidelines, all variants have been reclassified for interpreting sequence variants as P, LP, VUS, LB, or B [14]. The PM2 item in the ACMG classification is considered fulfilled if minor allele frequency (MAF) in relevant population databases is ≤ 0.1% [16]. The vast majority of reported pathogenic variants in arrhythmia and CM are extremely rare (MAF < 0.01%) [17]. The classification “high degree of pathogenicity” (item PVS1) should only be used for rare variants in genes where the loss of function is a well-established disease mechanism [18–20]. In the case of VUS, a rare variant classified as ambiguous does not provide molecular confirmation of a diagnosis. Still, it cannot be discarded as indicating a low risk of malignant arrhythmias for any patient, at least until additional data clarify its clinical role [21]. The VUS changed to LB due to a substantial increase of MAF seen with ongoing analysis of the global population, which notes the key role of global frequencies and its correlation with the prevalence of inherited arrhythmia and CM in the population [19]. Previous studies showed VUS rarely changed to P or LP variants [22].

### Sanger sequencing

The variant of *DSC2* p.I520T was screened again using Sanger sequencing (CX0020, TSINGKE Biological technology, Guangzhou, China) in the other members of the family. The mutation of *DSC2* p.K47Rfs*2 was screened again using the TA cloning technique in the other familial members. The purified PCR product was directly linked with pClone007 Versatile Simple Vector (TSINGKE Biological technology, Guangzhou, China) by TA cloning technique and transformed into Escherichia coli (DH5α). The extracted plasmids were sequenced with ABI 3730XL (Applied Biosystem, USA). The primer of TA cloning sequencing was as follows: M13F-47:5′-CGCCAGGGTTTTCCCAGTCACGAC-3′. The primer designed with Primer Premier 5.0 was used and showed as follows: forward primer, 5′-AAGGCTATTAGAAAGCAGAC-3′; reverse primer 5′-ATATGACCCAGAAACAAGAA-3′.

### Western blot

Tissues were homogenized on ice in the lysis buffer. After centrifugation and protein quantification, proteins were loaded onto SDS–PAGE gels and transferred onto nitrocellulose membranes. Membranes were incubated in 5% nonfat dry milk in TBST for 1 h at room temperature and incubated overnight at 4 °C with primary antibodies. Rabbit anti-DSC2 and mouse anti-β-actin antibody were purchased from Abcam Inc (Cambridge, MA). Antibodies were detected using 1:10,000 horseradish peroxidase-conjugated, donkey anti-rabbit, and donkey anti-mouse IgG (Jackson ImmunoResearch, USA). The Western blot luminol reagent was used to visualize bands corresponding to each antibody.

## Results

### Clinical presentation

**Fig. 1:**
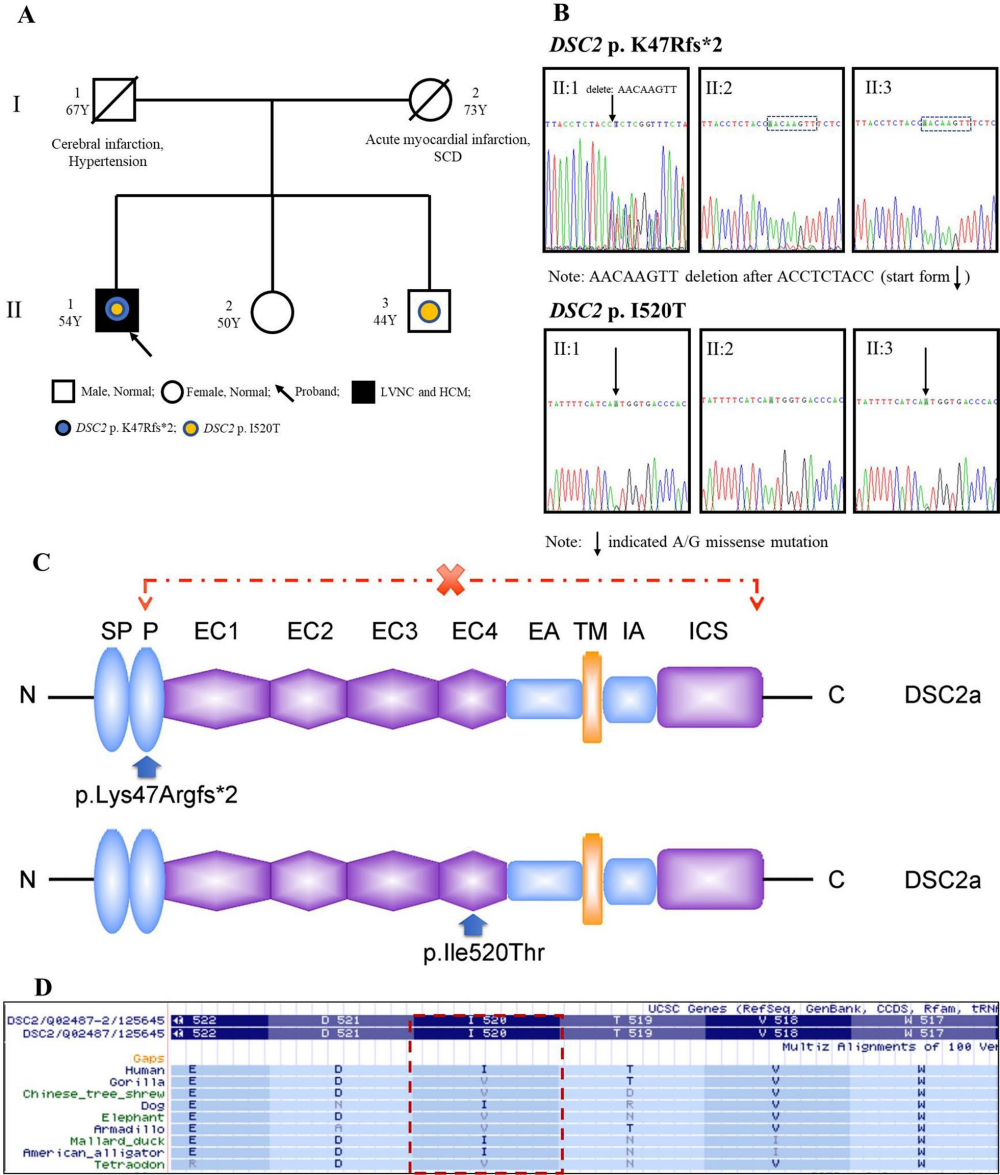
The familial pedigree, Sanger sequencing and the change of desmocollin2 protein. A: The pedigree of the family. II: 1 (the proband) presented with LVNC and HCM, complicating AF, VT and HF. SCD: sudden cardiac death. B: Sanger sequencing of *DSC2* variants in the family members. Sanger sequencing revealed that II: 1 carried two variants of *DSC2* p.K47Rfs*2 and p.I520T, which were not detected in II: 2. II: 3 only carried with *DSC2* p.I520T variant. C: the protein of desmocollin2 consists of several domains, including signaling peptide (S), proprotein (P), four extracellular cadherin domains (EC), extracellular anchor domain (EA), the transmembrane region (TM), the intracellular anchor (IA), as well as the intracellular cytoplasmic domain (ICS). X: the lost domains of desmocollin2 protein induced by *DSC2* p.K47Rfs*2 mutation. D: conservative analysis of *DSC2* p.I520

**Fig. 2:**
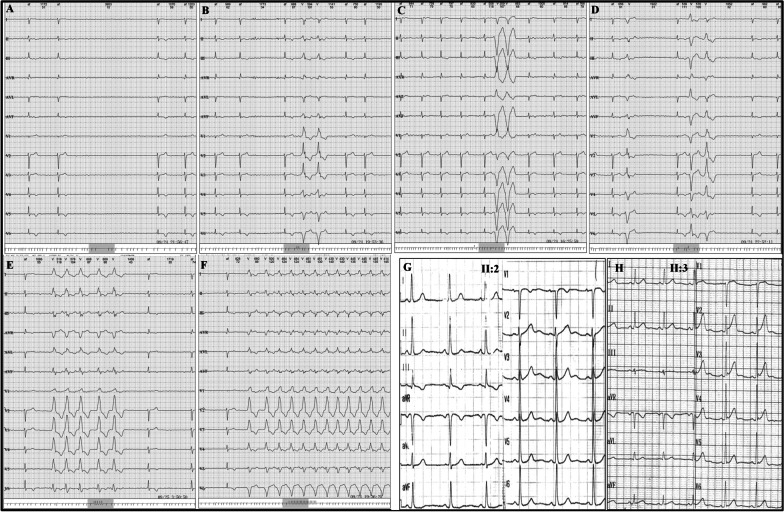
The electrocardiograms of familial members

The familial pedigree is shown in Fig. 1A. A male patient (proband, II: 1) aged 54 years was admitted to our hospital because of recurrent chest dullness and chest pain for three years and exacerbation for half a month. He suffered from poststernal chest distress and palm-sized chest pain, which were sometimes accompanied by palpitation. Symptoms usually occur during exhaustion and could be relieved after taking a break. The Holter of II: 1 (oral bisoprolol, 5 mg, once a day) showed persistent atrial fibrillation (AF) with a long RR interval of 3.95 seconds, low voltage in limb lead, and T wave inversion in leads of V_4_–V_6_ (Fig. 2A). The frequently paired premature ventricular extrasystoles (PVCs) originated from the left ventricular lateral wall (Fig. 2B), left ventricular apex (Fig. 2C), and left ventricular inflow tract (Fig. 2D). The episodes of ventricular tachycardia (VT) originated from the middle posterior septum (Fig. 2E) and lateral wall (Fig. 2F) of the left ventricle (LV). Coronary heart disease was excluded by coronary angiography. The level of NT-proBNP was 13,800.00 pg/ml (normal range: 0–125 pg/ml) during hospitalization and increased continuously during the follow-up.

**Fig. 3:**
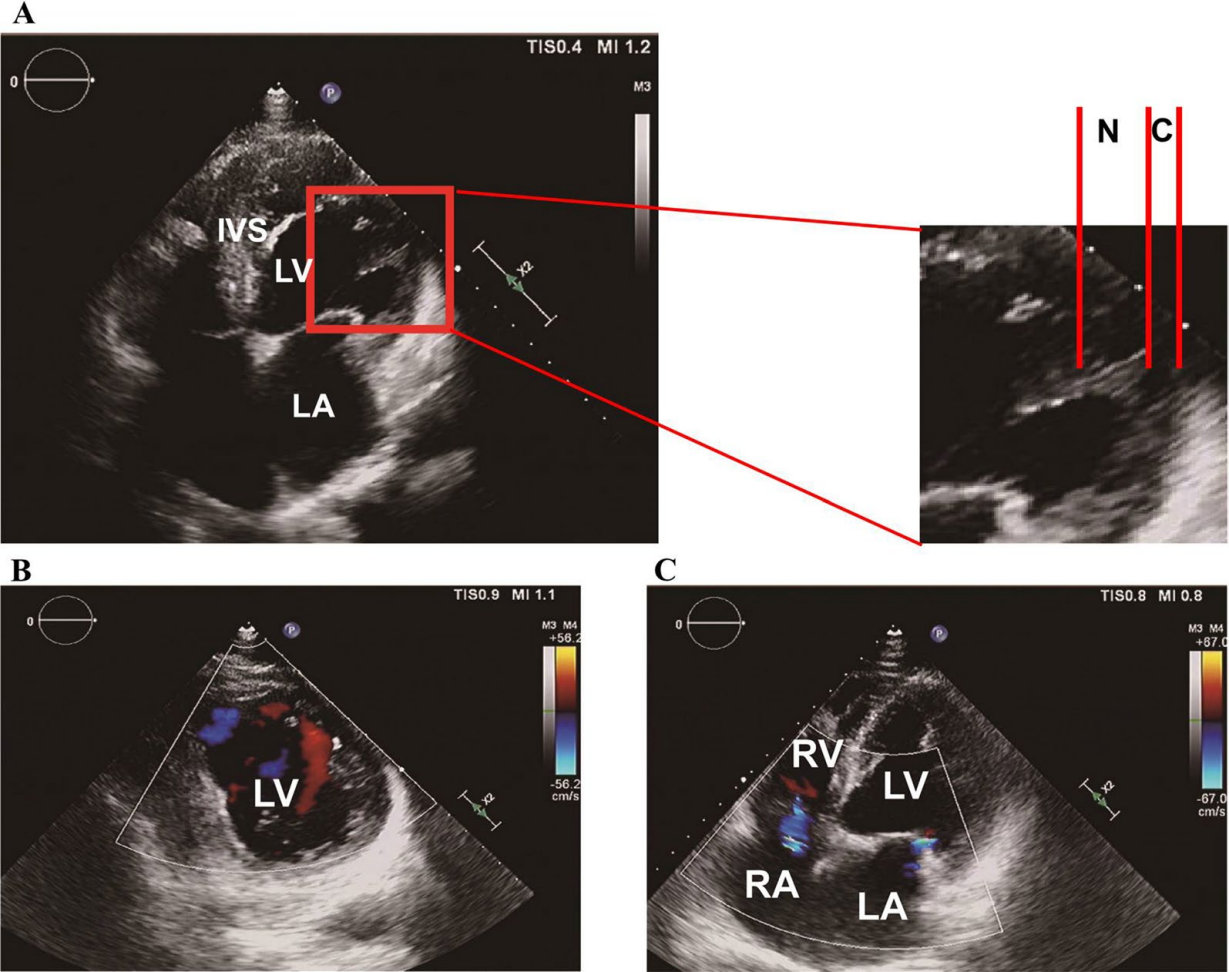
Echocardiographic characteristics of the proband II: 1. **A** To quantify the extent of noncompaction at the site of maximal wall thickness. The end-systolic ratio of noncompacted to compacted thickness was determined. The two layers were best visualized at end-systole as shown in this long-axis view (N, non-compacted layer; C, compacted layer). **B**, **C** Color Doppler study showed typical forward blood flow from the ventricular cavity into the deep spaces between the prominent trabeculations during diastole (in **B** represented by a red signal). Mild regurgitation could be seen in the mitral and tricuspid valves (**C**)

**Fig. 4:**
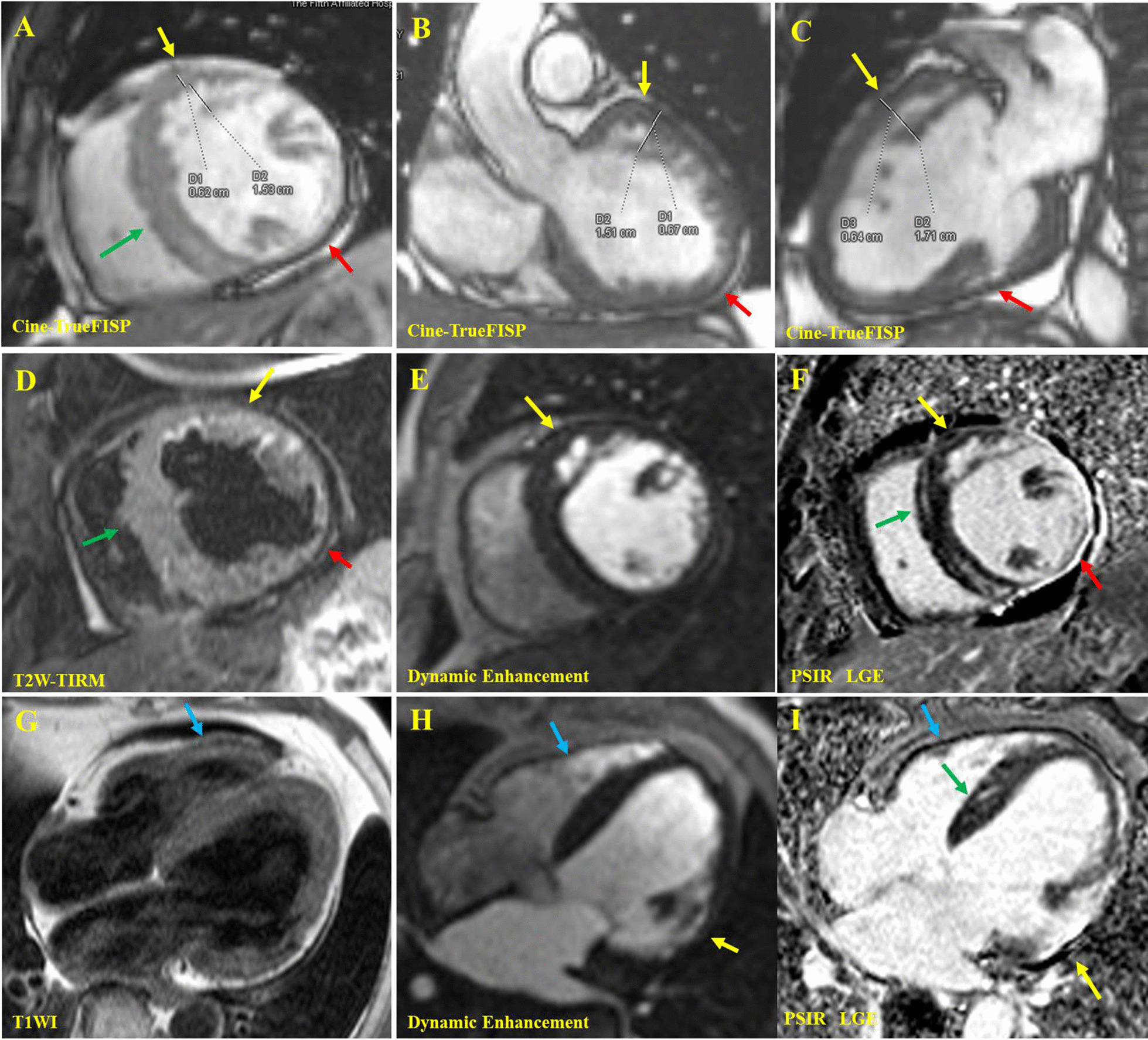
The cardiac magnetic resonance of the proband II: 1. In the end-diastolic images of cine True-FISP sequence, the short axis view of middle segment (**A**), coronal view of the left ventricular outflow tract (**B**), and three-chamber view of the heart (**C**) showed that myocardial thickening of the subendocardial, basal and middle segment, anterior and anterior-lateral wall of LV. The cardiac trabeculae increased and disordered, showing a reticular/palisade shape (yellow arrow). The maximum thickness ratio of the noncompacted layer to compacted layer (N/C = D2/D1) was between 2.25 and 2.67 in different sections. Deep recess was found among the trabeculae, and the communication existed between trabecular recess and the left ventricular cavity. The interventricular septum was thickened (green arrow) about 18 mm, and the inferior wall of LV became thinner (red arrow). The short-axis view in the middle segment (**D**) of the T2W-TIRM sequence showed thickening of the anterior and anterior-lateral wall of LV, increased signal intensity in the subendocardial region due to slow blood flow in trabecular recess (yellow arrow), localized thinning of the lateral-inferior wall (red arrow), and general thickening of the ventricular septum (green arrow). The short axis (**E**) and four-chamber (**G**, **H**) views of the first-pass enhancement sequence showed that the early enhancement signal of trabecular recess in the anterior and anterior-lateral wall of LV was consistent with that of the heart cavity (yellow arrow), indicating that there were flowing blood component in it. The short axis (**F**) and four-chamber (**I**) views of PSIR-LGE showed extensive abnormal enhancement in the lateral wall of LV (yellow arrow) and abnormal enhancement in the interventricular septum (green arrow). T1W showed no abnormal fat depositing signal in left and right ventricles (blue arrow). Yellow arrow: thickened lateral-anterior wall. Red arrow: thinned lateral wall. Green arrow: thickened interventricular septum. Blue Arrow: normal right ventricular wall

Echocardiography (Fig. 3) demonstrated the enlarged LV, thickened interventricular septum, thinning posterior wall of LV, and slightly enlarged right atrium. In addition, LV, especially the posterior and inferior walls, was hypokinetic and had an LVEF of 34%. The ratio of the non-compacted layer to compacted layer in LV was more than two folds at the end-systolic period of LV. The typical forward blood flowed from the ventricular cavity into the deep spaces between the prominent trabeculations during diastole. CMRI (Fig. 4) demonstrated that the ratio of the non-compacted layer to compacted layer at the end-diastolic period of LV was from 2.25 to 2.67. Deep recesses with slow blood flow could be seen among the trabeculae carneae, which communicated with the LV lumen, and this finding was consistent with the LVNC diagnosis. The basal segment, intermediate segment, anterior wall, and anterolateral wall of LV were thickened with increased, rough, and disorderly arranged trabeculae carneae in the subendocardial layer. The interventricular septum was thickened with a diameter of 18 mm. These changes suggested the combined phenotypes of LVNC and HCM. No apparent abnormal lipid signal deposition was observed in LV and right ventricle. II: 1 was administered with dabigatran (110 mg, orally, twice a day), fluvastatin (80 mg, orally, once each night), sakubatrovalsartan (50 mg, orally, twice a day), and trimetazidine (20 mg, orally, three times a day) for CMs, AF, VT, and HF therapy and metformin (50 mg, orally, three times a day) and carbamazin (100 mg, orally, once a day) for diabetes mellitus therapy since 10 years ago. II: 1 had the indication for implantable intracardiac defibrillator but refused implantation.

I: 1 (father of II: 1) suffered from hypertension and stroke. He was persistently laid in bed for three years and subsequently died at the age of 67 years. I: 2 (mother of II: 1) died of SCD induced by acute myocardial infarction on the way to the hospital at 73 years old. We could not receive clinical information and blood samples of I: 1 and I: 2. The echocardiograms of II: 2 and II: 3 were normal. No characteristic change related to CMs was observed in the ECGs of II: 2 and II: 3 (Fig. 2G–H).

### Genetic screening

We conducted genetic screening for the proband (II: 1) by target capture sequencing, and the rest of the family members were subjected to Sanger sequencing. The genetic screening revealed that the proband (II: 1) carried 10 exonic variants (Table 2), including two variants of *DSC2* p.K47Rfs*2 (NM_004949: EX2/CDS2: c.140_147delAACTTGTT) and *DSC2* p.I520T (NM_004949: EX11/CDS11: c.1559T>C), which were located in chromosome 18. The local and 1000 Genomes Project database frequency of *DSC2* p.K47Rfs*2 and p.I520T was less than 0.001. *DSC2* p.K47Rfs*2 was not detected in gnomAD exomes combined population. Desmocollin2 encoded by the *DSC2* gene was an important component for desmosome assembly. Abnormal desmocollin2 caused the dysfunction of cell–cell adhesions and intercellular gap junctions, failing to hold together the cardiomyocytes, fibrofatty myocardial replacement, cardiac conduction delay, mechanically ventricular dysfunction and arrhythmias [23]. According to the protein reference sequence, *DSC2* p.K47Rfs*2 converted the 47^th^ amino acid (AA) from lysine to arginine and led to a premature termination codon at the 48^th^ AA, which was therefore considered as frameshift and truncated mutation. This phenomenon caused the interrupting protein synthesis at the cadherin pro domain and loss of protein structure, including extracellular domains 1–4, extracellular anchor, a transmembrane domain, intracellular anchor, intracellular cadherin-like sequence, resulting in truncated protein without normal function (Fig. 1C). Based on the ACMG/AMP guidelines [14], the null variant (e.g., frameshift mutation) in a gene where the loss of function is a known disease mechanism was classified as PVS1, suggesting strong evidence of pathogenicity. Therefore, *DSC2* p.K47Rfs*2, as a truncated and loss-of-function mutation, served as PVS1. *DSC2* p.I520T (rs561310777) was demonstrated to be benign or likely benign by Clinvar database and the CHEO Genetics Diagnostic Laboratory in Children’s Hospital of Eastern Ontario (https://www.ncbi.nlm.nih.gov/clinvar/variation/155783/). The MAF of *DSC2* p.I520T was 0.02% and not fulfilled with the criteria of the vast majority of reported pathogenic variants (MAF < 0.01%). Additionally, according to the analysis of the UCSC Genome Browser, the amino acid site of *DSC2* p.I520 is not conserved in several species (Fig. 1D). Based on the genotype of II: 1, II: 2 did not carry heterozygous *DSC2* p.K47Rfs*2 or p.I520T, whereas II: 3 with normal electrocardiogram and echocardiography only carried the heterozygous *DSC2* p.I520T (Fig. 1A and B), indicating that these two variants were not linked on the same chromosome, but located on the homologous chromosomes, respectively. The variants of *CACNA1C*, *COL5A1*, *GDF1*, *HTRA1*, *MEF2A*, *TSPYL1*, and *TTN* were classified as benign or likely benign by the Clinvar database and ACMG guideline algorithm. Recently, no study confirmed that *ELN* variants cause CMs. The variant of *ELN* p.T248S was predicted to be tolerated/benign by SIFT and MetaSVM algorithms.

**Table 2:** Predisposing analysis of genetic variants suspected to arrhythmia and cardiomyopathies for II: 1

Chr	Gene	Transcript	Zygosity	RS-ID	1000G	Local Fre	GnomAD	SIFT	PolyPhen	MetaSVM	Clinvar	ACMG classification
chr12	*CACNA1C*	NM_001129827, c.5753C>T, p.T1918M	Het	rs201777030	0	A	0.0011	0.17 (T)	0.60 (P)	T	B	US
chr9	*COL5A1*	NM_000093, c.61C>T, p.P21S	Het	rs548525119	0	A	0.0011	0.43 (T)	0.00 (B)	T	B	B
chr18	*DSC2*	NM_004949, c.1559T>C, p. I520T	Het	rs561310777	0	A	0.0002	0.00 (D)	0.47 (P)	T	B	US
chr18	*DSC2*	NM_004949, c.140_147delAACTTGTT, p. K47Rfs*2	Het	–	0	A	–	–	–	–	–	LP
chr7	*ELN*	NM_000501, c.742A > T, p. T248S	Het	–	0	A	0.000004064	0.35 (T)	0.97 (P)	T	–	US
chr19	*GDF1*	NM_001492, c.470_471insGGC	Het	rs571387097	0	A	0.038	–	–	–	B	LB
chr10	*HTRA1*	NM_002775, c.59C > T, p.A20V	Het	rs369149111	0	A	0.0207	0.53 (T)	0.00 (B)	T	B	B
chr15	*MEF2A*	NM_005587, c.1234_1236delCAG	Het	rs373652230	0	A	0.1518	–	–	–	B	B
chr6	*TSPYL1*	NM_003309, c.528_529insGTG	Hom	rs397735194	0	A	–	–	–	–	–	B
chr2	*TTN*	NM_001267550, c.36655T>G, p. L12219V	Hom	rs139508281	0	A	0.0954	–	0.00 (U)	T	B	B

Chr: chromosome. Fre: frequency. Het: heterozygosis. Hom: homozygosis. GnomAD: frequency of existing variant in gnomAD exomes combined population. Local Fre: frequency information about this SNP in sequencing samples of over 200 normal people collected locally. Local frequency: 0–0.01 = A; 0.01–0.05 is B (including 0.01 and 0.05); 0.05–1 is C. P: possibly damaging; T: tolerated; U: unknown. 1000G: 1000 Genomes Project databases (2014version). B: benign. D: deleterious. US: uncertain significance. LB: likely benign. LP: likely pathogenic. –, no report

### Literature review of LVNC

We searched the NCBI database for studies with the theme of “left ventricle noncompaction” on June 13, 2021. We summarized the genes and their mutations associated with LVNC, which were predicted as likely pathogenic and pathogenic by ≥ 2 predicting algorithms, familial cosegregation validation, and molecular and/or animal/cell experiments, as shown in Table 3. Results showed that *MYH7*, *MYBPC3*, *TPM1*, *TAZ*, *TTN*, and *NONO* genes were the most common genes causing LVNC. LVNC caused by *MYH7* and *MYBPC3* mutations often complicated congenital heart diseases (CHD), such as atrial septal defect, ventricular septal defect, Ebstein, tetralogy of Fallot, patent ductus arteriosus, patent foramen ovale, and aortic hypoplasia. Some cases occurred with thromboembolism. Recently, LVNC induced by *DES* and *DSP* mutations was common and young-onset with HCM, DCM, ACM, myocarditis and HF, complicating ventricular and atrial tachycardias, and even needed therapy of heart transplantation in some cases. LVNC was associated with *FBN1* mutation combined with DCM and Marfan syndrome. LVNC induced by *HCN4* and *EMD* mutations complicated sick sinus syndrome (SSS), AF, atrial standstill, and interventricular block. Additionally, the LVNC induced by *EMD* mutation was combined with DCM. The LVNC caused by *RYR2*, *KCNQ1*, and *KCNH2* mutations occurred with catecholamine-sensitive VT, long QT syndrome (LQTs), torsade de pointes, and ventricular fibrillation. In contrast, the LVNC result from *SCN5A* mutation complicated SSS, AF, LQTs, Wolff–Parkinson–White syndrome, VT, and atrioventricular block. Some genes led to LVNC and caused complex and critical clinical disease syndromes, such as Rubinstein–Taybi syndrome (i.e., *ABCC9* gene), Ehlers–Danlos syndrome (i.e., *COL3A1* gene), Emery–Dreifuss muscular dystrophy (i.e., *EMD* gene), Danon disease (i.e., *LAMP2* gene), intellectual disability syndrome (i.e., *NONO* gene), Sotos syndrome (i.e., *NSD1* gene), LEOPARD syndrome (i.e., *PTPN11* gene), Coffin-Lowry syndrome (i.e., *RPS6KA3* gene), Cornelia de Lange syndrome (i.e., *SMC1A* gene), Barth syndrome (i.e., *TAZ* gene), and Holt–Oram syndrome (i.e., *TBX5* gene). Additionally, *ARFGEF2*, *MIPEP*, *NONO*, *SH2B1*, and *TMEM70* led to LVNC complicating developmental delay. The deletion of one of these genes, including *FKBP12*, *JARID2*, *NUMB*, and *PLZND1*, induced LVNC by affecting the activity of the Notch signaling pathway in animal models, which had not been discovered in clinical cases until now. For these genes from Table 3, only *ACTC1*, *ACTN2*, *DTNA*, *LDB3*, *MIB1*, *MYBPC3*, *MYH7*, *PRDM16*, *TNNT2*, and *TPM1* were reported to be associated with LVNC in the OMIM database (Table 4).

**Table 3:** The detailed mutations and their clinical characteristics associated with left ventricular noncompaction

Gene	Mutation	Sex	Aged	LVNC	HCM	DCM	ACM	RCM	HF	SCD/VF	CTR	MA	Other	FV	FA	
*ABCC9*	NM_005691, c.3594G>A, p.M1198I, rs199900459	M	32	+	−	−	−	−	−	−	−	−	Rubinstein–Taybi syndrome	−	−	[58]
*ACTC1*	NM_005159.4, c.62C>T, p.A21V	M	10 y	+	+	−	−	−	−	+	−	−	Transmural crypts	+	−	[59]
*ACTC1*	NM_005159.4, c.301G>A, p.E99K	M	11 y	+	+	−	−	−	−	−	−	−	−	+	+	[60]
*ACTC1*	NM_005159.4, c.478G>A, p.E101K	M	15 y	+	+	−	−	−	+	+	+	−	−	+	−	[61-64]
*ACTC1*	NM_005159.4, c.659A>C, p.Y220S	M	3 y	+	−	−	−	−	−	−	−	−	VT	−	−	[65]
*ACTC1*	NM_005159.4, c.692C>G, p.T231R	M	11 y	+	−	−	−	−	−	−	+	−	−	−	−	[65]
*ACTC1*	NM_005159.4, p.I289T	F	9 m	+	−	−	−	+	+	−	+	−	ASD	+	−	[66]
*ACTC1*	NM_005159.4, c.886T>C, p.Y296H	−	13 y	+	+	+	−	−	−	−	−	−	−	−	−	[67]
*ACTC1*	NM_005159.4, c.986T>C, p.I329T	F	48 y	+	−	−	−	−	−	+	−	−	VF, AF, IVB	+	−	[68]
*ACTC1*	NM_005159.4, c.670G>T, p.D224Y	−	−	+	−	−	−	−	−	−	−	−	−	−	−	[33]
*ACTC1*	NM_005159.4, c.281A>G, p.N94S	−	−	+	−	−	−	−	−	−	−	−	−	−	−	[33]
*ACTC1*	NM_005159.4, c.623G>A, p.R208H	−	−	+	−	−	−	−	−	−	−	−	−	−	−	[33]
*ACTN2*	NM_001103.2, c.683T>C, p.M228T	M	30 y	+	+	−	−	−	+	−	−	−	AF, AVB, esophageal atresia, tracheal fistula, ASD	+	−	[69]
*ACTN2*	chr1: 236881238:CCT>– NM_001278343, p.L70del	F	9 y	+	+	−	−	−	−	+	−	−		+	−	[9]
*ACTN2*	NM_001103.3, c.668T>C, p.L223P	M	15 y	+	+	+	−	−	+	−	−	−	−	+	−	[70]
*ANK2*	NM_001148.4, c.11150T>A, p.I3717N	−	−	+	−	−	−	−	−	−	−	−	−	−	−	[33]
*ANK2*	NM_001148.4, c.9145C>T, p.R3049W	−	−	+	−	−	−	−	−	−	−	−	−	−	−	[33]
*ALPK3*	NM_020778, c.639G>A, p.W213X	M	34 w	+	+	+	−	−	+	−	−	−	PFO, VSD	+	−	[71]
*ARFGEF2*	NM_006420.2, c.5126G>A, p.W1709X	F	10 y	+	−	−	−	−	−	−	−	−	Movement disorder, developmental delay and microcephaly	−	−	[72]
*BAG3*	NM_004281.3, c.465_466insGCG, p.A156dup	−	−	+	−	−	−	−	−	−	−	−	−	−	−	[33]
*BRAF*	chr7:140477829:->A, NM_004333, p.Q493H MYH6:chr14: 23858233:G>A, NM_002471, p.S1337L, rs758922922	M	9 y	+	+	+	−	−	+	−	−	−	AVB, SVT	−	−	[9]
*BMP10*	NM_014482.3, c.1219G>A, p.V407I	F	−	+	−	−	−	−	−	−	−	−	−	+	+	[73]
*BMP10*	deletion	−	−	+	−	−	−	−	−	−	−	−	−	−	+	[74]
*CACNA2D1*	g.81603880_81603881delAA, NM(-), NP(-), p.R652RfsX3 and RANGRF, NM(-), NP(-), p.P155S	F	1 m	+	−	−	−	−	+	+	+	−	Histiocytoid cardiomyopathy, WPW, arrhythmia storms	−	−	[75]
*CASQ2*	NM_001232.4, p.H244R	M	53 y	+	−	−	−	−	−	−	−	−	thrombus	+	−	[76]
*CASZ1*	NM(-), NP(-), c.2443_2459del, p.V1815PfsX14	M	11 m	+	−	+	−	−	+	−	−	−	−	−	−	[8]
*CDK10*	NM_052988.4, c.452T>C, p.L151P. Chromosome 16q24.3 deletion encompassing 9 genes: CDK10, CPNE7, DPEP1, CHMP1A, SPATA33, SPATA2L, VPS9D1, ZNF276,and FANCA	M	3 m	+	−	−	−	−	+	+	−	−	Partial agenesis of the corpus callosum, unilateral multicystic dysplastic kidney, a single central incisor with pyriform aperture stenosis	+	−	[77]
*COL3A1*	NM_000090.4, c.2959G>A, p.G987S	M	32 y	+	−	+	−	−	+	−	−	−	Vascular Ehlers-Danlos Syndrome, AF	−	−	[78]
*DES*	NM(-), NP(-), p.L69P	−	−	+	−	−	−	−	−	−	−	−	−	−	−	[79, 80]
*DES*	NM_001927.3, NP(-), c.336_344del, p.Q113_L115del	M	13 y	+	+	+	−	−	+	+	+	−	VT, AVB	+	+	[81]
*DES*	NM(-), NP(-), p.R212Q	−	−	+	−	−	−	−	−	−	−	−	−	−	−	[79, 80]
*DES*	NM(-), NP(-), p.A360S	−	−	+	−	−	−	−	−	−	−	−	−	−	−	[79, 80]
*DES*	NM(-), NP(-), c. C1360T, p.R454W	F	11 y	+	−	+	−	−	+	−	+	−	Coronary artery dissection, AVB, AFL	+	−	[82]
*DSC2*	NM_004949, c.140_147del, p. K47Rfs*2	M	54 y	+	+	−	−	−	+	−	−	−	AF, VT	+	−	This study
*DSC2*	NM_024422.3, c.1448A>T, p.N483I	−	−	+	−	−	−	−	−	−	−	−	−	−	−	[33]
*DSP*	NM(-), NC_000006.11, c.1339C>T	M	37 y	+	−	+	+	−	+	+	−	−	VT, myocarditis	+	−	[83]
*DSP*	NM_004415.2, c.3035delA, p.D1012fs	−	−	+	−	−	−	−	−	−	−	−	−	−	−	[33]
*DSP*	NM(-), NP(-), c.5208delAG, p.G1737fsX1742	M	5 y	+	−	+	−	−	+	+	−	−	Palmoplantar keratoderma	+	−	[84]
*DTNA*	NM(-), NP(-), c.146A>G, p.N49S	M	39 y	+	−	+	−	−	+	−	−	−	−	+	+	[85]
*DTNA*	NM(-), NP(-), c. 362 C>T, p.P121L	F	2 d	+	−	−	−	−	+	+	−	−	PDA, AF, VSD	+	−	[86-88]
*EMD*	NM(-), NP(-), c.226-2A>C	M	16 y	+	−	+	−	−	−	−	−	−	SSS, PFO, AS, Emery-deifuss muscular dystrophy	+	−	[89]
*EMD*	NM(-), NP(-), c.1A>G, p.M1V	M	53 y	+	−	+	−	−	+	−	−	−	SSS, AF, AS	+	−	[89]
*EMD*	NM(-), NP(-), c.23C>T, p.S8L	M	65 y	+	−	+	−	−	−	−	−	−	AVB, VT	+	−	[89]
*EMD*	NM(-), NP(-), c.415delC, p.L39fsX98	M	13 y	+	−	+	−	−	−	−	−	−	SSS, AS	+	−	[89]
*FBN1*	NM(-), NP(-), c.1633 C>T	F	14 y	+	−	+	−	−	+	−	−	−	Marfan syndrome	−	−	[90]
*FBN1*	NM(-), NP(-), c.3173 G>T	F	20 y	+	−	+	−	−	+	−	−	−	Marfan syndrome	−	−	[90]
*FBN1*	NM(-), NP(-), c.6832 C>T	M	2 y	+	−	+	−	−	+	−	−	−	Shprintzen-Goldberg	−	−	[90]
*FKBP12*	deletion	−	−	+	−	+	−	−	−	−	−	−	VSD, CHD	−	+	[38, 39]
*FKTN*	NM(-), NP(-), c.536G>C, p.R179T	M	26 y	+	−	+	−	−	+	−	−	−	−	−	−	[91]
*FLNC*	NM_001458.4, c.1933_1935del, p.645del	−	−	+	−	−	−	−	−	−	−	−	−	−	−	[33]
*FLNC*	NM(-), NP(-), c.4997T>C, p.I1666T	M	37	+	−	−	−	−	−	−	−	−	AF	−	−	[92]
*GATA4*	NM(-), NP(-), c.778 C>T, p.A242V and PTEN: NM(-), NP(-), c.517C>T, p.R173C	M	19 y	+	−	−	−	−	−	−	−	−	−	+	+	[93]
*HADHB*	NM(-), NP(-), c.1109+243_1438-703del	−	Fetus	+	+	−	−	−	+	+	−	−	TFP deficiency, lactic acidosis, hypoketotic hypoglycemia, and neonatal death	+	−	[94]
*HCN4*	NM_005477.2, c.1123C>T, p.R375C	F	16 y	+	−	−	−	−	−	−	−	−	SSS, left atrial dilatation	+	+	[33, 95]
*HCN4*	NM_005477.2, c.1231C>G, p.L411V	−	−	+	−	−	−	−	−	−	−	−	−	−	−	[33]
*HCN4*	NM(-), NP(-), c.1241C>G, p.A414G	M	74 y	+	−	−	−	−	−	−	−	−	SSS, AF	+	+	[96]
*HCN4*	NM_005477.2, c.1403C>T, p.A468V	−	−	+	−	−	−	−	−	−	−	−	−	−	−	[33]
*HCN4*	NM_005477.2, c.1438G>T, p.G480C	M	−	+	−	−	−	−	−	−	−	−	SSS	+	−	[33]
*HCN4*	NM(-), NP(-), c.1441T>C, p.Y481H	F	53	+	−	−	−	−	−	−	−	−	SSS, AF	+	+	[96]
*HCN4*	g.73622060 G>A, NM_005477.2, c. 1444G>A, p.G482R	M	23 y	+	−	−	−	−	+	+	−	−	SSS	+	+	[33, 96-98]
*HCN4*	NM(-), NP(-), p.R483_V487del	F	47 y	+	−	−	−	−	−	−	−	−	SSS, AF,Thoracic aortic aneurysms	+	−	[99]
*HCN4*	NM_005477.2, c.2432G>A, p.G811E	F	6 m	+	−	+	−	−	+	−	−	−	VSD, IVB	+	+	[100]
*HEY2*	NM_012259, c.683C>T p.T228M	−	−	+	−	−	−	−	−	−	−	−	−	−	−	[33]
*Jarid2*	deletion	−	−	+	−	−	−	−	−	−	−	−	VSD	−	+	[101]
*KCNH2*	NM_001204798, c.818 C>T, p.T273M	F	22 y	+	−	−	−	−	−	+	−	−	LQTs, Tdp, VT, VF	+	+	[102]
*KCNQ1*	NM(-), NP(-), c.817C>T, p.L273F	M	48 y	+	−	−	−	−	−	−	−	−	LQTs, VSD	+	−	[103]
*KCNQ1*	NM(-), NP(-), c.1831 G>T, p.D611T	F	5 y	+	−	−	−	−	−	+	−	−	LQTs, VT, VF, epilepsy	+	−	[104]
*LAMP2*	NM(-), NP(-), c.64+2T>A	F	23 y	+	−	+	−	−	+	−	−	−	VSD	+	−	[105]
*LAMP2*	NM_002294.2, c.987T>G, p.Y329X	M	21 y	+	−	−	−	−	−	−	−	−	Electrical myotonia, Danon disease	+	−	[106]
*LDB3*	NM_007078.2, c.608C>T, p.S203L	−	−	+	−	−	−	−	−	−	−	−	Congenital muscular dystrophy	−	−	[33]
*LDB3*	NM_007078.2, c.625G>C, p.E209Q	−	−	+	−	−	−	−	−	−	−	−	−	−	−	[33]
*LDB3*	NM(-), NP(-), c.163G>A, p.V55I	F	14 y	+	−	+	−	−	−	−	−	−	−	−	−	[88]
*LDB3*	NM(-), NP(-), c.349G>A, p.D117N	M	33 y	+	+	+	−	−	−	−	−	−	IVB	−	+	[107, 108]
*LDB3*	NM(-), NP(-), c.587C>T, p.S196L	M	40 y	+	+	+	−	−	+	+	−	−	−	+	+	[88, 109-111]
*LDB3*	NM(-), NP(-), c.1876G>A, p.D626N	M	13 y	+	−	−	−	−	−	+	−	−	WPW	+	−	[86, 88]
*LMNA*	NM(-), NP(-), c.1608+5G>C and LDB3: NM(-), NP(-), p.D117N	M	30 y	+	+	+	−	−	+	+	−	−	Limb girdle muscular dystrophy, VT, AF, CAVB	+	−	[76]
*LMNA*	NM(-), NP(-), c.1968+26A>G and TAZ: NM(-), NP(-), p.F128S	M	57 y	+	−	−	−	−	−	+	−	−	−	+	−	[76]
*LMNA*	NM_170707.2, c.738delG, p.Q246fs	−	−	+	−	−	−	−	−	−	−	−	−	−	−	[33]
*LMNA*	NC_000001, c. T1334A, p.V445E	M	23 y	+	−	−	−	−	−	+	−	−	VT/VF	+	+	[112]
*LMNA*	NM(-), NP(-), c.1930C>T, p.R644C	F	2 y	+	−	+	−	−	−	−	−	−	VSD, dysmorphism, multicystic and dysplastic kidney	+	−	[113, 114]
*MEF2A*	NM(-), NP(-), p.R17X	−	−	+	−	−	−	−	−	−	−	−	−	−	−	[80]
*MIB1*	NM(-), NP(-), c.1587C>T, p.R530X	−	−	+	−	−	−	−	−	−	−	−	−	+	+	[115]
*MIB1*	NM(-), NP(-), c.2827G>T, p.V943F	−	−	+	−	−	−	−	−	−	+		−	+	+	[115]
*MIB2*	NM_080875, c.2225T>G, p.V742G	F/M	−	+	−	−	−	−	−	−	−	−	Menetrier-like gastropathy	+	+	[116]
*MIB2*	NM_080875, c.2950G>C, p.V984L	F	−	+	−		−	−	−	−	−	−	Menetrier-like gastropathy	+	+	[116]
*MIPEP*	NM_005932, c.1745T>G, p.L582R and c.212T>A, p.L71Q	M	5.5 m	+	−	−	−	−	−	+	−	−	WPW, seizures, hypotonia, developmental delay, respiratory chain disorder	+	+	[117]
*MIPEP*	NM_005932, c.916C>T, p.L306F and c.1804G>T, p.E602X	F	11 m	+	−	+	−	−	−	+	+	+	Developmental delay, metabolic myopathy, diffuse neuronal loss, eosinophilic esophagitis	+	+	[117]
*MMACHC*	NM(-), NP(-), c.271dupA	F	35 w	+	−	−	−	−	−	+	−	−	Intracellular vitamin B12 disorder	−	−	[118]
*MYBPC3*	NM(-), NP(-), c.68G>A, p.G5R	M	20 y	+	−	−	−	−	−	−	−	−	−	−	−	[119]
*MYBPC3*	NM(-), NP(-), p.G148R	M	30 y	+	+	+	−	−	+	+	−	−	TOF, mesenteric thrombosis, myelofibrosis	+	−	[76]
*MYBPC3*	NM_000256.3, c.532G>A, p.V178M	−	−	+	+	−	−	−	−	−	−	−	−	−	−	[33]
*MYBPC3*	NM(-), NP(-), p.A216T and ACTC1: NM(-), NP(-), 22C>T	M	50 y	+	−	−	−	−	+	−	−	−	VT	+	−	[76]
*MYBPC3*	NM(-), NP(-), c.1523GA, p.G490R	M	32 y	+	−	−	−	−	−	−	−	−	−	+	−	[119]
*MYBPC3*	NM_000256.3, c.1504C>T, p.R502W	F	19 y	+	+	−	−	−	−	−	−	−	−	+	−	[33, 120]
*MYBPC3*	NM(-), NP(-), p.R502Q and p.R943X	M	−	+	+	−	−	−	−	+	+	−	−	+	−	[121]
*MYBPC3*	NM(-), NP(-), c.2373dup, p.W792fs	F	7 w	+	+	+	−	−	−	+	−	−	PFO and mitral valve insufficiency	+	−	[122]
*MYBPC3*	NM(-), NP(-), c.2460C>T, p.R820W	F	43 y	+	+	+	−	−	−	+	−	−	AF	+	−	[123]
*MYBPC3*	NM_000256, NP_000247, c.2572A>C, p.S858R	F	2 m	+	−	+	−	−	+	−	+	+	−	+	+	[124]
*MYBPC3*	NM(-), NP(-), c.2673C>T, p.P873L	M	37	+	−	−	−	−	+	−	−	−	Pulmonary hypertension	−	−	[119]
*MYBPC3*	NM(-), NP(-), c.2827C>T, p.R943X	M	5 w	+	+	+	−	−	+	+	−	−	ASD	+	−	[122]
*MYBPC3*	NM(-), NP(-), c.2919-2920delCT, p.P955RfsX95	F	28	+	−	−	−	−	−	−	−	−	VT	−	−	[119]
*MYBPC3*	NM(-), NP(-), c.2864_2865del, p.P955fs and c.1513_1515del, p.K505del	F	5 m	+	+	+	−	−	+	+	−	−	−	+	−	[125]
*MYBPC3*	NM(-), NP(-), c.2909G>A, p.R970Q	M	−	+	−	−	−	−	−	+	−	−	−	+	−	[65]
*MYBPC3*	NM(-), NP(-), c.3408C>A, p.Y1136X and c.2373dupG	M	36 w	+	+	−	−	−	+	−	−	−	−	+	−	[126]
*MYBPC3*	NM(-), NP(-), 377delA, p.Q1259fs and c.3599T>C, p.L1200P	M	11 d	+	+	−	−	−	+	+	+	+	−	+	−	[127]
*MLYCD*	c.393_400del8	F	10 m	+	−	+	−	−	−	−	−	−	Malonyl coenzyme A decarboxylase deficiency	−	−	[128]
*MYH6*	NM_002471.3, c.50G>T, p.R17L	−	−	+	−	−	−	−	−	−	−	−	CHD	−	−	[33]
*MYH6*	NM_002471.3, c.1793dupA, p.N598fs	−	−	+	−	−	−	−	−	−	−	−	−	−	−	[33]
*MYH6*	NM_002471.3, c.4828C>T, p.R1610C	−	−	+	−	−	−	−	−	−	−	−	−	−	−	[33]
*MYH7*	NM_000257, c.130C>T, p.Q44X and c.5029C>T, p.R1677C	M	32 y	+	−	−	−	−	+	−	−	−	Ebstein	+	−	[124]
*MYH7*	NM_000257, c.379C>A, p.P127T	−	−	+	−	−	−	−	−	−	−	−	−	−	−	[33]
*MYH7*	NM_000257, c.464_466del, p.delF155	M	8 y	+	−	−	−	−	−	−	−	−	ASD, Ebstein	−	−	[68]
*MYH7*	g.23901862T>G, NM_000257, p.Q163P	−	−	+	+	−	−	−	−	−	−	−	−	+	−	[129]
*MYH7*	NM_000257, c.801_803delGAC, p.D239del	M	65 y	+	−	−	−	−	+	−	−	−	AVB	−	−	[62]
*MYH7*	NM_000257, c.814G>A, p.R243H	M	25 y	+	−	−	−	−	+	−	+	−	AF	+	−	[62]
*MYH7*	NM_000257, c.745C>G, p.R249G	F	35 y	+	−	−	−	−	+	−	−	−	IVB	+	−	[68]
*MYH7*	NM_000257, c.840T>C, p.F252L	M	58 y	+	−	−	−	−	+	+	−	−	NSVT	−	−	[62]
*MYH7*	NM_000257, p.R281T	−	−	+	−	−	−	−	−	−	−	−	Ebstein, CHD	+	−	[130]
*MYH7*	NM_000257, p.Y283D	F	49 y	+	−	−	−	−	−	−	−	−	Ebstein, ASD, VSD, CHD	+	−	[130, 131]
*MYH7*	NM_000257, c.847T>C, p.Y283H	M	−	+	−	−	−	−	−	−	−	−	−	+	−	[65]
*MYH7*	NM_000257, p.L301Q	M	12 y	+	−	−	−	−	+	−	−	−	Ebstein	+	−	[76, 130]
*MYH7*	NM_000257, p.Y350D	−	−	+	−	−	−	−	−	−	−	−	Ebstein	−	−	[130]
*MYH7*	NM_000257, p.E1350del	F	32 y	+	−	−	−	−	+	−	−	−	Aortic insufficiency	+	−	[76]
*MYH7*	NM_000257, p.Y350N	F	26 y	+	−	−	−	−	−	−	−	−	Ebstein	−	−	[131]
*MYH7*	NM_000257, c.1085T>G, p.M362R	F	infant	+	−	−	−	−	−	−	−	−	Ebstein, ASD, VSD	+	−	[132, 133]
*MYH7*	NM_000257, c.1106G>A, p.R369Q	F	8 m	+	−	+	−	−	+	−	−	−	−	+	−	[127, 68]
*MYH7*	NM_000257, p.L390P	M	59 y	+	−	−	−	−	−	−	−	−	Ebstein, PFO, AF, CHD	−	−	[131, 130]
*MYH7*	NM_000257, c.1207C>T, p.R403W and c.1000–1G>A	M	14 y	+	+	−	−	−	−	+	−	−	VF	+	−	[124]
*MYH7*	NM_000257, p.K1459N	−	−	+	−	−	−	−	−	−	−	−	Ebstein	−	−	[130]
*MYH7*	g:23897795G>C, NM_000257, c. 1492C>G, p.Q498E	M	19 y	+	−	−	−	−	−	−	−	−	−	+	−	[134]
*MYH7*	NM_000257, c.1678T>G, p.M531R	F	14 y	+	−	+	−	−	+	+	−	−	−	+	+	[135, 136]
*MYH7*	NM_000257, p.D545N and p.D955N	M	35 y	+	−	−	−	−	+	−	−	−	Thrombus	+	−	[76]
*MYH7*	g.23896462G>A, NM_000257, p.S648L	−	−	+	−	+	−	−	−	−	−	−	−	+	−	[129]
*MYH7*	NM_000257, p.L658V	M	61 y	+	+	−	−	−	−	−	−	−	−	−	−	[76]
*MYH7*	NM_000257, c.A2010_G2031del, p.R671_E677del	M	11 y	+	−	−	−	−	+	−			VSD	−	−	[137]
*MYH7*	g.23895236T>C, NM_000257, p.E700G	−	−	+	+	−	−	−	−	−	−	−	−	+	−	[129]
*MYH7*	NM_000257, c.2155C>T, p.R719W	M	29 y	+	+	−	−	−	+	+	−	−	−	+	−	[138]
*MYH7*	NM_000257, c.2419C>G, p.R807G	−	−	+	−	−	−	−	−	−	−	−	−	−	−	[33]
*MYH7*	NM_000257, p.I818N	M	21 y	+	+	−	−	−	−	+	−	−	−	+	−	[76]
*MYH7*	NG_016984.1, p.R890C	F	1 m	+	−	+	−	−	+	−	−	−	PDA, PFO	+	−	[139]
*MYH7*	NM_000257, p.C905R	M	30 y	+	−	+	−	−	−	−	−	−	Cardiac valvular disease	+	−	[140]
*MYH7*	NM_000257, c.2785G>A, p.E929K	M	42 y	+	−	+	−	−	+	+	−	−	VT, IVB	+	−	[68]
*MYH7*	NM_000257, c.3586C>T, p.H1196Y	−	−	+	−	−	−	−	−	−	−	−	−	−	−	[33]
*MYH7*	NM_000257, p.1220delE	M	35 y	+	−	−	−	−	−	−	−	−	Ebstein	−	−	[130, 131]
*MYH7*	NM_000257, c.3830G>C, p.R1277P	−	−	+	−	−	−	−	−	−	−	−	−	−	−	[33]
*MYH7*	NM_000257, c.3748C>T, R1250W	M	55 y	+	+	+	−	−	+	−	+	−	−	+	−	[127]
*MYH7*	NM_000257, p.R723G and p.S1335L	M	22 y	+	+	−	−	−	−	+	+	−	−	+	−	[121]
*MYH7*	NM_000257, c.4161C>T, p.R1359C	M	29 y	+	−	−	−	−	+	−	−	−	AF	−	−	[62]
*MYH7*	NM_000257, c.4090G>C, p.A1364P	M	−	+	−	−	−	−	−	−	−	−	−	+	−	[65]
*MYH7*	NM_000257, p.K1459N	F	58 y	+	−	−	−	−	−	−	−	−	Ebstein, SVT	−	−	[131]
*MYH7*	NM_000257, p.Y1488C	M	41 y	+	−	−	−	−	+	−	−	−	−	+	−	[76]
*MYH7*	NM_000257, c.4588C>T, p.R1530X	−	−	+	−	−	−	−	−	−	−	−	−	−	−	[33]
*MYH7*	NM_000257, p.E1573K	F	33 y	+	−	−	−	−	−	−	−	−	Ebstein, VSD	+	−	[130, 131]
*MYH7*	NM_000257, c.5030G>A, p.R1677H	M	−	+	−	−	−	−	−	−	−	−	−	+	−	[65]
*MYH7*	NM_000257, c.5382G>A, p.A1766T	M	20 y	+	−	−	−	−	−	−	−	−	−	−	−	[62]
*MYH7*	NM_000257, c.5401G>A, p.E1801K	M	25 y	+	−	−	−	−	−	−	−	−	AVB, neurological deficits, Laing distal myopathy	+	−	[141]
*MYH7*	NM_000257, c.5566G>A, p.E1856K	F	37 y	+	−	+	−	−	+	+	−	−	VF, distal myopathy	+	−	[142]
*MYH7*	NM_000257, p.N1918K	M	5 y	+	−	−	−	−	−	−	−	−	Ebstein, aorta coarctation	+	−	[131, 130, 76]
*MYH7*	NM_000257, p.R1925G	F	50 y	+	−	−	−	−	+	+	−	−	−	+	−	[76]
*MYH7*	NM_000257, c.8183G>C	M	14 y	+	−	−	−	−	−	−	−	−	−	+	−	[62]
*MYLK2*	NM_033118.3, c.1754T>A, p.I585N	−	−	+	−	−	−	−	−	−	−	−	−	−	−	[33]
*MYLK2*	NM_033118.3, c.1658G>A, p.R553H	−	−	+	−	−	−	−	−	−	−	−	−	−	−	[33]
*MYOT*	NM_000257, c.179C>T	M	72 y	+	−	−	−	−	−	−	−	−	Myopathy	−	−	[143]
*MYPN*	NM_032578.2, c.3457G>A, p.G1153R	−	−	+	−	−	−	−	−	−	−	−	−	−	−	[33]
*NEBL*	NM_006393.2, c.2747C>T, p.P916L	M	37 y	+	−	+	−	−	+	−	−	−	VT	−	−	[144]
*NKX2.5*	NM(-), NP(-), p.K183N	F	30 y	+	−	+	−	−	+	+	−	−	ASD, VF	+	−	[145]
*NKX2.5*	NM(-),NP(-), c.512InsGC, p.170X	M	−	+	−	−	−	−	−	+	−	−	ASD, conduction defects,	+	+	[146]
*NKX2.5*	NM_004387.3, c.604C>G, p.L202V	−	−	+	−	−	−	−	−	−	−	−	−	−	−	[33]
*NEXN*	NM_144573.3, c.2012T>C, p.I671T	−	−	+	−	−	−	−	−	−	−	−	−	−	−	[33]
*NEXN*	NM_144573.3, c.1396A>C, p.K466Q	−	−	+	−	−	−	−	−	−	−	−	−	−	−	[33]
*NNT*	NM_012343, c.638_639insT, p.R213fs	−	−	+	−	+	−	−	+	−	+	+	−	+	+	[129]
*NONO*	NM_001145408.1, c.154+9A>G. MYH6: NM_002471.3, c.718G>A, p.D240N	−	Fetus	+	−	−	−	−	−	−	−	−	Mitral valve dysplasia, and aorta hypoplasia	+	+	[147]
*NONO*	NM_001145408, c.1171+1G>T	M	17 y	+	−	−	−	−	−	−	−	−	Intellectual disability syndrome	−	−	[148]
*NONO*	NM_001145408, c.1171+1G>A	M	4 y	+	+	−	−	−	−	−	−	−	Intellectual disability syndrome	−	−	[149, 150]
*NONO*	NM_001145408.1, the first three coding exons and 19 kb of sequence upstream of the transcriptional start site	M	1 m	+	−	−	−	−	+	+	+	−	PFO, Ebstein, developmental delay, encephalopathy, seizures and dysautonomia, cerebellum dysplasia	+	−	[151]
*NONO*	NM_001145408, c.154+5_154+6delGT, p.N52SfsX6	M	2 y	+	−	+	−	−	+	−	−	−	Ebstein, PFO, intellectual disability syndrome	+	−	[152]
*NONO*	NM_001145408.1, c.246_249del, p.P83TfsX7	M	fetus	+	−	−	−	−	−	−	−	−	Ebstein, PS, VSD, VSD, PLSVA, cardiovascular hypoplasia or transposition	+	−	[153]
*NONO*	NM_001145408, c.550C>T, p.R184X	M	2 y	+	−	+	−	−	−	−	−	−	ASD, VSD, PDA, aortic dilatation, intellectual disability syndrome	−	−	[149, 150]
*NONO*	NM_001145408, c.1093C>T, p.R365X	M	10 y	+	+	+	−	−	+	−	−	−	RVH, ASD, VSD, PDA, Intellectual disability syndrome	−	−	[151]
*NONO*	NM_001145408, c.1394dupC, p.N466KfsX13	M	5 y	+	−	−	−	−	−	−	−	−	ASD, VSD, PDA, intellectual disability syndrome	−	−	[151]
*NONO*	NM_001145408.1, c.471del, p.Q157HfsX18	M	fetus	+	−	−	−	−	−	+	−	−	Ebstein, PS, ASD, VSD, aortic arch variation, endocardial fibroelastosis, PFO, corpus callosum hypoplasia			[153]
*NRG1*	NM(-), NP(-), c.661G>A, p.W143X	M	−	+	−	−	−	−	−	−	−	−	−	+	+	[73]
*NSD1*	NM(-), NP(-), c.6218_6219insG	M	11 y	+	−	+	−	−	−	+	−	−	Sotos syndrome	−	−	[1]
*NSD1*	NM(-), NP(-), c.2604_2605dupTT	F	6 y	+	+	−	−	−	−	−	−	−	Sotos syndrome	−	−	[1]
*NUMB*	deletion	−	−	+	−	−	−	−	−	−	−	−	VSD	−	+	[154]
*OBSCN*	g.228552766_228552767delinsG, NM(-), NP(-), p.T7266RfsX53	M	56 y	+	−	−	−	−	−	−	−	−	−	−	−	[155]
*OBSCN*	g.228559442delC, NM(-), NP(-), p.S7947PfsX82, rs71180793	F	30 y	+	−	−	−	−	−	−	−	−	−	−	−	[155]
*OBSCN*	g.228562285G>C, NM(-), NP(-), c.25367-1G>C, rs55883237	M	39 y	+	−	−	−	−	−	−	−	−	−	−	−	[155]
*PDLIM3*	NM_014476.4, c.742C>T, p.R248C	−	−	+	−	−	−	−	−	−	−	−	−	−	−	[33]
*PKP2*	deletion	F	12 d	+	−	−	−	−	+	+	−	−	−	+	−	[50]
*PKP2*	NM_004572.3, c.2018G>A, p.G673D	−	−	+	−	−	+	−	−	−	−	−	−	−	−	[33]
*PLEKHM2*	NM(-), NP(-), c.2156_2157delAG, p.K645AfsX12	M	7 y	+	−	+	−	−	+	+	+	−	VT	+	+	[156]
*Plxnd1*	deletion	−	−	+	−	−	−	−	−	−	−	−	VSD, aortic arch anomalies, persistent truncus arteriosus	−	+	[42]
*PLN*	NM(-), NP(-), p.R14del	F	48 y	+	−	−	−	−	−	−	−	−	MVT, thrombus	+	−	[76]
*PRDM16*	NM_022114.3, c.1047dupC, p.S350fsX48	M	4 m	+	−	+	−	−	−	−	−	−	−	+	−	[157]
*PRDM16*	g.3322083C>T, NC_000001.10, c.1057C>T, p.Q353X	−	31 w fetus	+	−	+	−	−	−	−	−	−	−	+	−	[158]
*PTPN11*	NM(-), NP(-), p.W279C	M	40 y	+	+	−	−	−	−	−	−	−	AF, LEOPARD syndrome	−	−	[159]
*RBM20*	NM_001134363, c.1901 G>T, p.R634L	F	39 y	+	−	−	−	−	−	+	+	−	TOF	+	+	[34]
*RBM20*	NM_001134363, c.1907G>A, p.R636H	F	11 y	+	−	+	−	−	+	−	−	−	−	+	−	[33, 160]
*RBM20*	NM_001134363, c.1909A>G, p.S637G	M	13 y	+	−	+	−	−	+	−	−	−	−	+	−	[160]
*Rac1*	deletion	−	−	+	−	−	−	−	−	−	−	−	VSD, CHD	−	+	[161]
*RPS6KA3*	NM(-), NP(-), c.1000-2A>G	M	1 y	+	−	+	−	+	+	+	−	−	Coffin–Lowry syndrome	+	−	[162]
*RYR2*	deletion	F	20 y	+	−	+	−	−	−	+	−	−	AF, VF	+	−	[163-165]
*RYR2*	NM(-), NP(-), c.506G>A, p.R169Q	F	5 y	+	−	−	−	−	−	+	−	−	CPVT, VF	+	+	[30]
*RYR2*	NM_001035.2, c.169-?_c.273+?del	M	−	+	−	−	−	−	−	−	−	−	−	+	−	[33]
*RYR2*	NM_001035.2, c.878A>C, p.Q293P	−	−	+	−	−	−	−	−	−	−	−	−	−	−	[33]
*RYR2*	NM_001035.2, c.6180G>T, p.Q2060H	−	−	+	−	−	−	−	−	−	−	−	−	−	−	[33]
*RYR2*	NM_001035.2, c.13936G>C, p.D4646H	−	−	+	−	−	−	−	−	−	−	−	−	−	−	[33]
*RYR2*	NM(-), NP(-), p.I4855M	F	10 y	+	−	−	−	−	−	+	−	−	CPVT, ASD	+	+	[166]
*SCN5A*	NM_198056.1, c.1141-3C>A	M	13 y	+	−	−	−	−	+	−	−	−	PVC, WPW	+	−	[167]
*SCN5A*	NM_198056.1, c.87G>A, rs6599230	M	43 y	+	−	−	−	−	−	−	−	−	PVC, LQTs	+	−	[167]
*SCN5A*	NM_198056.1, c.453C>T	F	1 w	+	−	−	−	−	+	−	−	−	PVC	+	−	[167]
*SCN5A*	NM_198056.1, c.1673A>G, p.H558R, rs1805124	M	13 y	+	−	−	−	−	+	−	−	−	PVC, WPW	+	−	[167]
*SCN5A*	NM_198056.1, c.3269C>T, p.P1090L, rs1805125	M	4 y	+	−	−	−	−	+	−	−	−	AF, SSS, PVC	+	−	[167]
*SCN5A*	NM_198056.1, c.3996G>A	M	0 y	+	−	−	−	−	+	−	−	−	AVB	−	−	[167]
*SCN5A*	NM_198056.1, c.5457T>C, rs1805126	F	5 y	+	−	−	−	−	+	−	−	−	PSVT, VT, AVB, LQTs, SSS, AF, WPW	+	−	[167]
*SDHD*	NM(-), NP(-), c.275A>G, p.D92G	M	neonate	+	+	−	−	−	−	+	−	−	Mitochondrial complex II deficiency	−	−	[168]
*SH2B1*	Deletion	M	18 d	+	−	−	−	−	−	−	−	−	Aorta stenosis, ASD, developmental delay	+	−	[169]
*SLC39A8*	Deletion	−	−	+	−	−	−	−	−	−	−	−	−	−	+	[170]
*SMC1A*	NM(-), NP(-), c.1636_1638delATT	F	21 m	+	+	−	−	−	−	−	−	−	Microform Cleft Lip, poor Vision, Cornelia de Lange Syndrome	+	−	[171]
*STRA6*	NM_022369.4, c.113+3_113+4del	−	Fetus (22 w)	+	−	−	−	−	−	−	−	−	Syndromic microphthalmia, interrupted aortic arch type A	+	−	[172]
*TAZ*	NM_000116.3, p.R94H	M	12 h	+	−	−	−	−	+	+	−	−	−	+	−	[173]
*TAZ*	NM_000116.3, intron1+9G>C	M	4 m	+	−	+	−	−	+	+	−	−	Barth syndrome	+	−	[174]
*TAZ*	NM_000116.3, c.IVS8-1G>C	M	3 m	+	+	+	−	−	+	+	−	−	−	+	−	[86] [88]
*TAZ*	NM_000116.3, IVS10+2T>A	M	8 m	+	−	−	−	−	+	+	−	−	Barth syndrome	+	−	[87, 110]
*TAZ*	NM_000116.3, c.109+1G>C	M	4 m	+	−	+	−	−	+	+	−	−	Barth syndrome	+	−	[175]
*TAZ*	NM_000116.3, c.777+2T>A	M	infant	+	−	+	−	−	+	+	−	−	Barth syndrome	−	−	[176]
*TAZ*	NM_000116.3, c.134_136delinsCC, p.H45PfsX38	M	1.0	+	−	−	−	−	+	+	−	−	Barth syndrome	+	−	[177]
*TAZ*	NM_000116.3, 158InsC, p.L53Pfs80X	M	19 y	+	−	−	−	−	−	−	−	−	Barth syndrome	−	−	[88]
*TAZ*	chrX:153641550:T>C, NM_000116, p.L82P	M	1 m	+	−	+	−	−	+	+	−	−	VT, VF	−	−	[9]
*TAZ*	NM_000116.3, p.C118R	−	5 m	+	−	−	−	−	+	+	−	−	−	−	−	[178]
*TAZ*	NM_000116.3, p.C118R and p.T352C	M	5 m	+	−	+	−	−	+	−	−	−	Barth syndrome			[87, 110]
*TAZ*	NM_000116.3, c.367C>T, p.R123X	M	20 y	+	−	−	−	−	−	+	−	−	Barth syndrome	+	−	[177]
*TAZ*	NM_000116.3, c.527A>G, p.H176R	M	3.0 y	+	−	−	−	−	+	+	−	−	Barth syndrome	+	−	[177]
*TAZ*	NM_000116.3, c.583G>T, p.G195X	M	45 y	+	−	+	−	−	+	−	+	−	AF, ASD, barth syndrome	+	−	[179]
*TAZ*	NM_000116.3, p.G197R	−	0 d	+	−	−	−	−	+	+	−	−	−	−	−	[178]
*TAZ*	NM_000116.3, c.646G>A, p.G216R	M	14 m	+	−	−	−	−	+	−	−	−	Barth syndrome	+	+	[180]
*TAZ*	chrX:153648583:A>insAA, NM_000116, p.Y227_F228delinsX	M	6 m	+	+	+	−	−	+	+	−	−	−	−	−	[9]
*TAZ*	NM_000116.3, c.710_711delTG, p.V237AfsX73	M	6.5 y	+	−	−	−	−	−	+	−	−	Barth syndrome	+	−	[177]
*TAZ*	chrX: 153649305:G>-, NM_000116, p.A281QfsX58	M	1 y	+	+	+	−	−	−	+	−	−	−	−	−	[9]
*TBX5*	NM_000192.3, c.510+5G>T	F	34 y	+	−	−	−	−	−	−	−	−	SSS, AF, ASD, Holt-Oram syndrome	+	−	[181]
*TBX5*	NM_000192.3, p.S36Tfs*25	F	49 y	+	−	+	−	−	−	−	−	−	SSS, Holt-Oram syndrome	+	−	[181]
*TBX5*	NM(-), NP(-), c.791G>A, p.R264K	M/F	3 m	+	−	+	−	−	+	+	−	−	Embolism, VSD	+	+	[182]
*TBX20*	NM(-), NP(-), c.785C>T, p.T262M	F	−	+	−	−	−	−	−	−	−	−	−	+	+	[183]
*TBX20*	NM(-), NP(-), c. 951C>A, p.Y317X	M/F	−	+	−	+	−	−	−	−	+	−	−	+	+	[183]
*TMEM43*	NM_024334.2, c.317A>G, p.Y106C	−	−	+	−	−	−	−	−	−	−	−	−	−	−	[33]
*TMEM70*	NM_017866.5, c.141delG, p.P48Rfs*2, and c.316+1G>A	M	36 w	+	+	−	−	−	−	−	−	−	Developmental delay, undescended testicle	+	+	[184]
*TNNC1*	NM(-), NP(-), c.243G>C, p.M81I	F	5 m	+	−	−	−	−	−	−	−	−	−	+	−	[65]
*TNNC1*	NM(-), NP(-), c.281A>C, p.E94A	F	4 m	+	−	−	−	−	+	−	+	−	−	−	−	[65]
*TNNC1*	NM(-), NP(-), c.304C>T, p.R102C	F	12 y	+	−	−	−	−	−	−	+	−	−	+	−	[65]
*TNNI3*	NM_000363.4, c.575C>A, p.R192H	F	13 y	+	+	−	−	−	−	+			−	−	−	[185]
*TNNT2*	NM_001001430.1, c.?GAG>AAG, p.E96K	F	5 m	+	−	−	−	−	+	+	+	−	−	+	−	[186]
*TNNT2*	NM_000364.3, c.305G>A, p.R102Q	F	44 y	+	+	−	−	−	−	−	−	−	AF, IVB	+	−	[68]
*TNNT2*	NM(-), NP(-), p.D117N	F	45 y	+	+	−	−	−	−	−	−	−	SVT	+	−	[76]
*TNNT2*	NM_001001431, c.450C>T, p.R131W	−	−	+	−	−	−	−	−	−	−	−	−	−	−	[62, 187]
*TNNT2*	NM(-), NP(-), p.K210del	M	14 y	+	−	+	−	−	+	−	+	−	−	+	−	[121]
*TPM1*	NM_00101805.1, c.41A>G, p.D14G	F	20 d	+	−	−	−	−	+	+	−	−	−	−	−	[65]
*TPM1*	NM(-), NP(-), c.109A>G, p.K37E	F	8 y	+	−	−	−	−	−	+	−	−	VF, SCD	+	−	[65, 188]
*TPM1*	NM_001018007, c.377C>G, p.L113V	F	22 w	+	−	−	−	−	+	+	+	+	Mitral valve insufficiency, pulmonary hypertension	+	−	[189]
*TPM1*	NM 001018005.1, c.475G>A, p.D159A	F	2 y	+	−	−	−	−	+	+	+	−	Ebstein	−	−	[190]
*TPM1*	NM_00101805.1, NP_001018005.1, c.533G>A, p.R178H	F	13 d	+	−	−	−	−	+	−	+	−	−	−	+	[65]
*TPM1*	NM(-), NP(-), c.765G>A, p.E192K	M	55 y	+	−	−	−	−	−	−	−	−	−	−	−	[119]
*TPM1*	NM_001018020.1, c.725C>T, p.A242V	M	45 y	+	−	+	−	−	−	+	−	−	VT, AF, IVB	+	−	[68]
*TPM1*	NM(-), NP(-), c.933A>G, p.K248E	M	63 y	+	−	−	−	−	+	−	−	−	−	+	−	[119]
*TPM1*	g.23857430C>C, NM(-), NP(-), p.D275H	−	−	+	−	+	−	−	−	−	−	−	−	+	−	[129]
*TTN*	NM(-), NP(-), c.533C>A, p.A178D	M	20 y	+	+	+	−	−	+	+	−	−	−	+	+	[191]
*TTN*	NM(-), NP(-), c.8858_8859del, p.F2953fs	M	17 y	+	−	−	−	−	+	−	−	−	−	+	−	[34]
*TTN*	NM_001256850.1, c.43360C>T, p.R14454X	−	−	+	−	−	−	−	−	−	−	−	CHD	−		[33]
*TTN*	NM_001256850.1, c.44248C>T, p.R14750X	−	−	+	−	−	−	−	−	−	−	−	−	−	−	[33]
*TTN*	NM_001256850.1, c.53947C>T, p.R17983X	−	−	+	−	−	−	−	−	−	−	−	−	−	−	[33]
*TTN*	NM(-), NP(-), c. 54668G>A, p.G18223D	F	30 y	+	−	−	−	−	−	−	−	−	Embolism	+	−	[34]
*TTN*	NM_001256850.1, c.61961G>A, p.W20654X	−	−	+	−	−	−	−	−	−	−	−	−	−	−	[33]
*TTN*	NM_001256850.1, c.64100_64101insTTGA, p.D21368X	−	−	+	−	−	−	−	−	−	−	−	−	−	−	[33]
*TTN*	NM_001256850.1, c.80845C>T, p.R26949X	−	−	+	−	−	−	−	−	−	−	−	CHD	−	−	[33]
*TTN*	NM(-), NP(-), c.81307_81310del, p.I27103fs	M	16 y	+	−	−	−	−	+	+	+	−	AF, VT	+	−	[34]
*TTN*	NM_001256850.1, c.82724delA, p.N27575fs	−	−	+	−	−	−	−	−	−	−	−	−	−	−	[33]
*TTN*	NM(-), NP(-), c.83889_83890del, p.Y27963fs	M	52 y	+	−	−	−	−	+	−	+	−	AF, VT	+	−	[34]
*TTN*	chr2: 179425207: GGAACTGTAAATG>-, NM_001267550, p.28547QfsX12, rs762286447	M	1 y	+	−	+	−	−	+	−	−	−	AVB, ASD	+	−	[9]
*TTN*	NM_001256850.1, c.93376delA, p.R31126fs	−	−	+	−	−	−	−	−	−	−	−	−	−	−	[33]
*TTN*	NM_001256850.1, c.98039_98040insTCAA, p.N32680fs	−	−	+	−	−	−	−	−	−	−	−	−	−	−	[33]
*TTN*	NM(-), NP(-), c. 9388+1G>C, p.E2989EfsX4 and c. 102439T>C, p.W34072R	F	38 y	+	−	+	−	−	+	−	+	−	VSD, arthrogryposis multiplex congenital	+	−	[192]
*YWHAE*	NM(-), NP(-), c.-458G>T	M	2 w	+	−	−	−	−	−	+	−	−	Hypoplasia of the corpus callosum	+	+	[193]
Mitochondrial DNA																
*Met31*	m. 3397A>G	−	−	+	−	−	−	−	−	−	−	−	−	−	−	[194]
*Met31*	m. 3398T>C	M	35 y	+	−	−	−	−	−	−	−	−	−	−	−	[194]
*ND1*	m.3308T>C	F	6 y	+	−	+	−	−	−	−	−	−	Ebstein	+	+	[195, 196]

M: male. F: female. y: years. w: weeks. m: months. d: days. LVNC: left ventricular noncompaction. HCM: hypertrophic cardiomyopathy. DCM: dilated cardiomyopathy. ACM: arrhythmogenic cardiomyopathy. RCM: restricted cardiomyopathy. HF: heart failure. SCD: sudden cardiac death. FV: family verification. FA: functional analysis. CTR: cardiac transplantation. MA: mechanical assist. fs: frame-shift mutation. X: truncated mutation. VT: ventricular tachycardia. VF: ventricular fibrillation. MVT: ventricular tachycardia needed resuscitation. AVB: atria-ventricular block. AF: atrial fibrillation. AS: atrial standstill. IVB: intra-ventricular block. ASD: atrial septal defect. CHD: congenital heart disease. VSD: ventricular septal defect. SVT: supraventricular tachycardia. PFO: patent foramen ovale. TOF: fallot tetralogy. SVT: supraventricular tachycardia. PVC: premature ventricular contraction. LQTs: long QT syndrome. SSS: sick sinus syndrome. WPW: Wolff–Parkinson–White syndrome. TdP: torsade de pointes. CPVT: Catecholamine sensitive ventricular tachycardia. PDA: patent ductus arteriosus. PLSVA: persistent left superior vena cava. PS: pulmonary stenosis. RVH: right ventricular hypertrophy. −, not mentioned in the previous reports. +, mentioned/occurred in previous reports

**Table 4:** The phenotypes of genes associated with left ventricular noncompaction in OMIM database

Location	Genes	Full name	Gene/locus MIM number	Phenotypes in OMIM
12p12.1	*ABCC9*	ATP binding cassette subfamily C member 9	601439	AF; DCM; hypertrichotic osteochondrodysplasia
15q14	*ACTC1*	Actin alpha cardiac muscle 1	102540	ASD; DCM; HCM; LVNC
1q43	*ACTN2*	Actinin alpha 2	102573	DCM; HCM; LVNC; myopathy
4q25-q26	*ANK2*	Ankyrin 2	106410	Cardiac arrhythmia; LQTs
15q25.3	*ALPK3*	Alpha kinase 3	617608	HCM
20q13.13	*ARFGEF2*	ADP ribosylation factor guanine nucleotide exchange factor 2	605371	Periventricular heterotopia with microcephaly
10q26.11	*BAG3*	BAG cochaperone 3	603883	DCM; myofibrillar myopathy
7q34	*BRAF*	B-Raf proto-oncogene, serine/threonine kinase	164757	Adenocarcinoma of lung, somatic; cardiofaciocutaneous syndrome; colorectal cancer, somatic; LEOPARD syndrome; melanoma, malignant, somatic; nonsmall cell lung cancer, somatic; Noonan syndrome
2p13.3	*BMP10*	Bone morphogenetic protein 10	608748	–
7q21.11	*CACNA2D1*	Calcium voltage-gated channel auxiliary subunit alpha2delta 1	–	–
1p13.1	*CASQ2*	Calsequestrin 2	114251	CPVT
1p36.22	*CASZ1*	Castor zinc finger 1	609895	–
2q32.2	*COL3A1*	Collagen type III alpha 1 Chain	120180	Ehlers–Danlos syndrome, vascular type; polymicrogyria with or without vascular type Ehlers–Danlos syndrome
2q35	*DES*	Desmin	125660	DCM; myofibrillar yopathy; Scapuloperoneal syndrome, neurogenic, kaeser type
18q12.1	*DSC2*	Desmocollin2	125645	ACM; mild palmoplantar keratoderma and woolly hair
6p24.3	*DSP*	Desmoplakin	125647	ACM; DCM; woolly hair and keratoderma; keratoderma and tooth agenesis; epidermolysis bullosa, lethal acantholytic; keratosis palmoplantaris striata II; Skin fragility-woolly hair syndrome
18q12.1	*DTNA*	Dystrobrevin alpha	601239	LVNC; CHD
Xq28	*EMD*	Emerin	300384	Emery-Dreifuss muscular dystrophy
15q21.1	*FBN1*	Fibrillin 1	134797	Acromicric dysplasia; ectopia lentis, familial; geleophysic dysplasia; marfan lipodystrophy syndrome; Marfan syndrome; MASS syndrome; Stiff skin syndrome; Weill–Marchesani syndrome
20p13	*FKBP12*	FKBP prolyl isomerase 1A	186945	–
9q31.2	*FKTN*	Fukutin	607440	DCM; muscular dystrophy-dystroglycanopathy
7q32.1	*FLNC*	Filamin C	102565	HCM; RCM; distal myopathy; myofibrillar myopathy
2p23.3	*HADHB*	Hydroxyacyl-CoA dehydrogenase trifunctional multienzyme complex subunit beta	143450	Trifunctional protein deficiency
15q24.1	*HCN4*	Hyperpolarization activated cyclic nucleotide gated potassium channel 4	605206	Brugada syndrome; SSS
6q22.31	*HEY2*	Hes related family bHLH transcription factor with YRPW motif 2	604674	–
6p22.3	*JARID2*	Jumonji and AT-rich interaction domain containing 2	–	–
8p23.1	*GATA4*	GATA binding protein 4	600576	Testicular anomalies with or without congenital heart disease; ASD; VSD; TOF
11p15.5-p15.4	*KCNQ1*	Potassium voltage-gated channel subfamily Q member 1	607542/604115	LQTs; SQTs; AF; Jervell and Lange–Nielsen syndrome; Beckwith–Wiedemann syndrome
7q36.1	*KCNH2*	Potassium voltage-gated channel subfamily H member 2	152427	LQTs
Xq24	*LAMP2*	Lysosomal associated membrane protein 2	309060	Danon disease
10q23.2	*LDB3*	LIM domain binding 3	605906	DCM; HCM; LVNC; myofibrillar myopathy
1q22	*LMNA*	lamin A/C	150330	DCM; RCM; Charcot-Marie-Tooth disease; Emery-Dreifuss muscular dystrophy; Heart-hand syndrome; Hutchinson-Gilford progeria; lipodystrophy; Malouf syndrome; mandibuloacral dysplasia; muscular dystrophy
15q26.3	*MEF2A*	Myocyte enhancer factor 2A	600660	Coronary artery disease
1p34.1	*MMACHC*	Metabolism of cobalamin associated C	609831	Methylmalonic aciduria and homocystinuria
18q11.2	*MIB1*	MIB E3 ubiquitin protein ligase 1	608677	LVNC
1p36.33	*MIB2*	MIB E3 ubiquitin protein ligase 2	611141	–
13q12.12	*MIPEP*	Mitochondrial intermediate peptidase	602241	Combined oxidative phosphorylation deficiency
11p11.2	*MYBPC3*	Myosin binding protein C3	600958	DCM; HCM; LVNC
16q23.3	*MLYCD*	Malonyl-CoA decarboxylase	606761	Malonyl-CoA decarboxylase deficiency
14q11.2	*MYH7*	Myosin heavy chain 7	160760	DCM; HCM; LVNC; laing distal myopathy; myopathy, myosin storage; Scapuloperoneal syndrome
20q11.21	*MYLK2*	Myosin light chain kinase 2	606566	HCM
5q31.2	*MYOT*	Myotilin	604103	Myofibrillar myopathy; myopathy, spheroid body
10p12.31	*NEBL*	Nebulette	605491	–
5q35.1	*NKX2.5*	NK2 homeobox 5	600584	ASD; AVB; conotruncal heart malformations; Hypoplastic left heart syndrome; hypothyroidism, congenital nongoitrous; TOF; VSD
1p31.1	*NEXN*	Nexilin F-actin binding protein	613121	DCM; HCM
5p12	*NNT*	Nicotinamide nucleotide transhydrogenase	607878	Glucocorticoid deficiency with or without mineralocorticoid deficiency
Xq13.1	*NONO*	Non-POU domain containing octamer binding	300084	Mental retardation
8p12	*NRG1*	Neuregulin 1	142445	Schizophrenia
5q35.3	*NSD1*	Nuclear receptor binding SET domain protein 1	606681	Sotos syndrome
1q42.13	*OBSCN*	Obscurin, cytoskeletal calmodulin and titin-interacting RhoGEF	–	–
4q35.1	*PDLIM3*	PDZ and LIM domain 3	–	–
12p11.21	*PKP2*	Plakophilin 2	602861	ACM
1p36.21	*PLEKHM2*	Pleckstrin homology and RUN domain containing M2	609613	–
3q22.1	*PLXND1*	Plexin D1	604282	–
6q22.31	*PLN*	Phospholamban	172405	HCM; DCM
1p36.32	*PRDM16*	PR/SET domain 16	605557	DCM; LVNC
12q24.13	*PTPN11*	Protein tyrosine phosphatase non-receptor type 11	176876	LEOPARD syndrome; leukemia, juvenile myelomonocytic, somatic; metachondromatosis; Noonan syndrome
10q25.2	*RBM20*	RNA binding motif protein 20	613171	DCM
Xp22.12	*RPS6KA3*	Ribosomal protein S6 kinase A3	300075	Coffin–Lowry syndrome; mental retardation
1q43	*RYR2*	Ryanodine receptor 2	180902	ACM; CPVT
3p22.2	*SCN5A*	Sodium voltage-gated channel alpha subunit 5	600163	Sudden infant death syndrome; AF; Brugada syndrome; DCM; LQTs; SSS; VF
11q23.1	*SDHD*	Succinate dehydrogenase complex subunit D	602690	Mitochondrial complex II deficiency; araganglioma and gastric stromal sarcoma; paragangliomas with or without deafness
16p11.2	*SH2B1*	SH2B adaptor protein 1	608937	–
4q24	*SLC39A8*	Solute carrier family 39 member 8	608732	Congenital disorder of glycosylation
Xp11.22	*SMC1A*	Structural maintenance of chromosomes 1A	300040	Cornelia de Lange syndrome; developmental and epileptic encephalopathy, with or without midline brain defects
15q24.1	*STRA6*	Signaling receptor and transporter of retinol STRA6	610745	Microphthalmia with coloboma; Microphthalmia, syndromic
X A7.3; X 37.95 cM	*TAZ*	Tafazzin	300394	Barth syndrome
12q24.21	*TBX5*	T-box transcription factor 5	601620	Holt–Oram syndrome
7p14.2	*TBX20*	T-box transcription factor 20	606061	ASD
3p25.1	*TMEM43*	Transmembrane protein 43	612048	ACM; Emery-Dreifuss muscular dystrophy
8q21.11	*TMEM70*	Transmembrane protein 70	612418	Mitochondrial complex V (ATP synthase) deficiency, nuclear type
3p21.1	*TNNC1*	Troponin C1, slow skeletal and cardiac type	191040	DCM; HCM
19q13.42	*TNNI3*	Troponin I3, cardiac type	191044	DCM; RCM; HCM
1q32.1	*TNNT2*	Troponin T2, cardiac type	191045	DCM; RCM; HCM; LVNC
15q22.2	*TPM1*	tropomyosin 1	191010	DCM; HCM; LVNC
2q31.2	*TTN*	Titin	188840	DCM; HCM; muscular dystrophy, limb-girdle; myofibrillar myopathy with early respiratory failure; salih myopathy; tibial muscular dystrophy, tardive
17p13.3	*YWHAE*	Tyrosine 3-monooxygenase/tryptophan 5-monooxygenase activation protein epsilon	605066	–

LVNC: left ventricular noncompaction. HCM: hypertrophic cardiomyopathy. DCM: dilated cardiomyopathy. ACM: arrhythmogenic cardiomyopathy. RCM: restricted cardiomyopathy. AF: atrial fibrillation. ASD: atrial septal defect. CHD: congenital heart disease. VSD: ventricular septal defect. PFO: patent foramen ovale. TOF: fallot tetralogy. LQTs: long QT syndrome. SQTs: short QT syndrome. SSS: sick sinus syndrome. WPW: Wolff–Parkinson–White syndrome. CPVT: catecholamine sensitive ventricular tachycardia. VF: ventricular fibrillation. PDA: patent ductus arteriosus. –, not mentioned in OMIM database

### Expression of DSC2

**Fig. 5:**
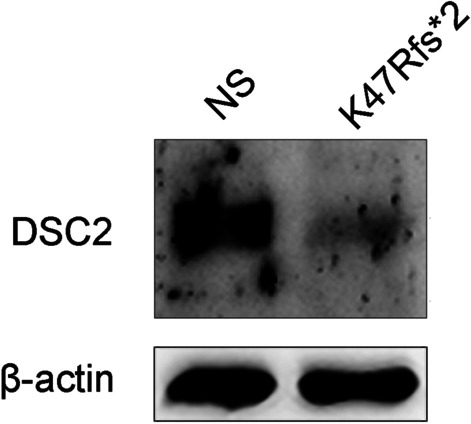
The expression of desmocollin2. NS: normal control (the healthy volunteer). The samples of NS and *DSC2* p.K47Rfs*2 were collected from the skin and subcutaneous tissue in the upper left limb of the healthy volunteer and the proband (II: 1)

The western blot (Fig. 5) showed that the expressing level of functional desmocollin2 protein (~ 94kd) was lower in the proband than that in the healthy volunteer, indicating that *DSC2* p.K47Rfs*2 remarkably and abnormally reduced the functional desmocollin2 expression in the proband.

## Discussion

In our study, we have first discovered that the truncated mutation (p.K47Rfs*2) of *DSC2* remarkably and abnormally reduced the functional desmocollin2 expression, as an important component of desmosome assembly, which may consequently induce rare overlap phenotypes of LVNC and HCM, complicating AF, VT, and HF.

LVNC is a rare primary CM and serves as a global cardiac disease induced by the arrest of normal embryogenesis of the endocardium and mesocardium that leads to prominent trabeculations and deep intertrabecular recesses within the LV wall communicating with the cavity, a thin compacted epicardial layer, and acquired ventricular wall remodeling [24]. The inferior and lateral walls of LV from the midcavity to the apex are the most commonly involved [10]. The Jenni and Petersen diagnosis criteria of LVNC are generally accepted and rely on imaging modalities, including echocardiography and CMRI, e.g., (1) bilayer myocardium with multiple, prominent trabeculations in the end-systole; (2) NC/C ratio of > 2:1 in echocardiography or > 2.3 in CMRI measured in the end-systole or end-diastole, respectively; and (3) communication with the intertrabecular space and deep intertrabecular recesses in the noncompaction layer [10, 11]. Based on previous studies [25] and our literature review, the LVNC prevalence is 0.05%–0.3% in an adult population undergoing echocardiography. LVNC may occur with congenital heart malformations, myopathies, neuromuscular and metabolic diseases, complicating developmental delay and intellectual disability. LVNC with HF, ventricular arrhythmias, and thromboembolic events show poor prognosis with an overall mortality rate of 5–12% per year [26]. As shown in the literature review in our study, once the cases occur with LVNC and malignant clinical syndromes, the prognosis is poor with high mortality in the fetus or infant. Some LVNC patients have a family history of LVNC and/or sudden death and recurrent VT despite therapy with multiple antiarrhythmic drugs. The ventricular arrhythmia ordinarily originates from bilateral ventricles. The substrate of ventricular arrhythmia in LVNC typically involves the mid-apical segment, septum, and lateral wall of LV. In contrast, focal PVCs commonly arise from LV basal-septal regions and/or papillary muscles, reflecting the distribution of noncompaction myocardial segments [27]. LVNC mostly affects the left ventricular muscle and serves as a primary and genetically heterogeneous CM, which the American Heart Association exists in sporadic and hereditary forms. Previous research showed that among LVNC cases, 52% are sporadic, 32% have a genetic cause, and an additional 16% have affected family members with negative genetic testing. Not all family members present with the same LVNC phenotype as some proband occurs with DCM, HCM, or SCD [28]. Potentially causative gene mutations responsible for LVNC are involved in the sarcomere, cytoskeleton, mitochondria, desmosome, and storage and ion channels implicated in other cardiac diseases [29–31]. According to previous studies and our literature review, *MYH7*, *MYBPC3*, *TPM1*, *TAZ*, *TTN*, and *NONO* genes are the most common pathogenic mutations in LVNC [28, 32, 33], which are not demonstrated in the proband of our study. LVNC or an element of other CMs may be isolated due to sharing common genetic risk [34]. These common genetic backgrounds are phenotypically expressed as overlap CMs, fulfilling the criteria for LVNC and HCM (or DCM, RCM, or ACM). The LV hypertrabeculation and HCM may present as acquired conditions due to the adaptation of the ventricular myocardium to pressure or volume overload [35]. Some LVNCs also involve the right ventricle, exhibiting high values of right ventricular noncompaction and trabeculated area [36]. Therefore, the subtypes of LVNC include benign LVNC, LVNC with arrhythmias, dilated LVNC, hypertrophic LVNC, hypertrophic dilated LVNC, restrictive LVNC, right ventricular or biventricular LVNC, and LVNC with CHD [25]. Actually, some pathogenic mutations are associated with various complex rare diseases involving more than one organ. For example, the 11-16^th^ exon deletion of *KCNQ1* induced mild Beckwith-Wiedemann and severe LQTs [37]. Notably, in light of our literature review, LVNC with complex clinical syndromes, poor prognosis, and high mortality that may occur in the fetus or infant should be classified as another new subtype of LVNC. These syndromes may include Rubinstein–Taybi syndrome, Ehlers–Danlos syndrome, Emery–Deifuss muscular dystrophy, Danon disease, intellectual disability syndrome, Soto’s syndrome, LEOPARD syndrome, Coffin-Lowry syndrome, Cornelia de Lange syndrome, Barth syndrome, and Holt–Oram syndrome.

Two independent biological events leading to abnormal trabeculations and compaction, including hypertrabeculation and noncompaction, are demonstrated by the *FKBP12*-deficient mouse model for LVNC [38, 39]. Hypertrabeculation refers to the phenotype with increased number and thickness of the trabeculae at the embryonic stage. In contrast, noncompaction refers to the lack of trabecular remodeling toward the compact wall during and after trabeculations [40]. The enhanced Notch signaling induces abnormal cardiomyocyte proliferation and hypertrabeculation/noncompaction via Neuregulin1, EphrinB2, FKBP12, BMP10, TBX20 and its upstream of Sema3E/PlexinD1 signaling pathway, which potentially contributes to LVNC [41, 42]. The inhibition of Notch signaling partly normalizes the abnormal hypertrabeculation phenotype in *FKBP12*-deficient hearts. The planar cell polarity (PCP) signaling is also an essential molecular mechanism of polarity establishment for epithelial cells in planes orthogonal to the apical-basal axis. The noncanonical Wnt signaling, which is independent of β-catenin, is critical for PCP signaling. Together, they serve as the Wnt/PCP signaling pathway, which is composed of core components (i.e., Frizzled, Disheveled, Prickle, Vangl, and Celsr1), PCP regulator (i.e., casein kinase 1ε) and a large number of PCP effectors (i.e., Daam1, Rac1, and PhoA) [43, 44]. These molecules of Wnt/PCP signaling induced by their genetic mutations result in abnormal polarization, organization of cardiomyocyte in ventricular and outflow myocardia, and subsequent LVNC pathogenesis [40].

In this study, the proband (II: 1) carried DSC2 p.K47Rfs*2 and p.I520T variants. According to the ACMG classification criteria, the *DSC2* p.K47Rfs*2 is a truncated and loss-of-function mutation, likely to cause LVNC and HCM. Compared to the MAF (< 0.01%) of inherited arrhythmia and CMs in the population, the MAF of *DSC2* p.I520T (0.02%) is relatively high. Moreover, *DSC2* p.I520T is not conservative in multiple species. In addition, the ECG is the main tool to judge the early changes of CMs. Especially, the abnormal changes of ECG caused by CMs are often earlier than that of the cardiac structure illustrated by echocardiography [45]. Whereas II: 3 only carrying *DSC2* p.I520T and without cardiac event showed normal ECG and echocardiography. Therefore, we speculated that *DSC2* p.I520T might not be pathogenic. In fact, as an important component of the cardiac desmosome, desmocollin2 maintains the normal electromechanical connection among cardiomyocytes. The desmocollin2 abnormality would lead to various CMs (such as DCM and HCM), HF, complex arrhythmia and even SCD. Recent research and our literature review showed that the abnormal components of the cardiac desmosome, including *DES* and *DSP* genes, are associated with LVNC. Desmosome is the intracellular structure that anchors intermediate filament to the plasma membrane. Desmosome, adherent junction, and gap junction are the major components of the intercalated disc, which is vital for the cell–cell adhesion and electronic coupling of cardiomyocytes [46]. Desmocollin2 and desmoglein2 (encoded by *DSG2*) are the important familial members of cadherin. Desmocollin2 and desmoglein2 form the adhesive heterodimer, which provides a structural framework for desmosome assembly together with armadillo proteins (plakoglobin and plakophilin, which are encoded by *PG* and *PKP2*, respectively) and the plakin family (desmoplakin, which DSP encodes) [47, 48]. The desmin filament (encoded by *DES*), also expressed in cardiac desmosomes, connects Z-bands, costameres, and nuclei with the cytoskeleton. The pathogenic mutations associated with desmosome proteins may cause desmosome dysfunction and remodel intercalated disc, leading to the disturbance of the mechanical–electrical coupling of cell–cell adhesion and further alteration of signaling pathways.

Previous studies show that the homozygous deletion mutation of *PKP2* lead to LVNC and rapid HF, whereas the heterozygous *DES* p.A337P (NM_001927.3, c.1009G>C) induces LVNC and skeletal myopathy [49, 50]. A report indicates a *DSC2* variant (NM_024422.3, c.1448A>T, p.N483I) in a patient with sporadic LVNC, but whether the variant is the cause of LVNC remains unclear [32]. A previous study shows that the *DSC2* deletion in zebrafish induces the reduction of the desmosome plaque area, deficiency of desmosome extracellular electron-dense midlines, and disturbance of myocardial contractility [51]. Additionally, *DSC2* p.Q554X (c.1660C>T) participates in the pathogenicity of familial ACM [52]. *DSC2* p.A897fs*900 shows an obvious reduction in desmosome binding to plakoglobin and plakophilin [53]. The truncated mutation of *DSC2* (c.2553delA) decreases the connexin 43 expression and lost plakoglobin signal [54]. Furthermore, the loss of *DSC2* activates the AKT/β-catenin pathway. AKT inhibition suppresses β-catenin-dependent transcription and proliferation, which are caused by *DSC2* knockdown [55]. In the present study, the 47th AA lysine of the *DSC2* protein sequence at the cadherin pro is replaced by arginine, leading to premature codon termination. Consequently, *DSC2* p.K47Rfs*2 remarkably and abnormally reduced the functional desmocollin2 expression, which may interfere with desmosome formation and the stability of the intermediated disc, and disturb the mechanical stress and electrical signal propagation of cardiomyocytes. Notably, the pathological and imaging changes induced by *DSC2* p.K47Rfs*2 are observed in LV, resulting in the subendocardial thickening of the anterior lateral wall and interventricular septum, and thinning in part of the left ventricular wall. The ECG of the proband showed low voltage in the limb leads but no typical ST-T change, commonly suggesting diffuse myocardial lesion. The VT arises from the middle-posterior septum and lateral wall of LV. PVCs originate from the lateral wall and apex of LV and the left ventricular inflow tract, and this finding is consistent with the cardiac lesion extent. Additionally, trabeculae are observed with serious dysplasia and distributed in a rough and disordered pattern. An apparent slow blood flow is observed in the recess of trabeculae, which may increase the risk of thrombus. However, the mechanism of *DSC2* p.K47Rfs*2 that induces overlap phenotypes of LVNC and HCM remains unknown. There is no research about the relationship among *DSC2*, Notch, and Wnt/PCP signaling pathways.

## Limitation

The limitations of the present study are as follows. First, the molecular mechanism of *DSC2* p.K47Rfs*2 leading to abnormalities of the desmosome structure and function should be further explored. Second, *DSC2* p.K47Rfs*2 is the main cause of LVNC and HCM but still cannot rule out *DSC2* p.I520T, or other variants may increase the risk of LVNC and HCM phenotype penetrance. Third, the mechanism of how the *DSC2* p.K47Rfs*2 induces overlap phenotypes needs further research. Fourth, the parents and grandparents of the patient (II: 1) could not be tracked because of death. The II: 1, II: 2 and II: 3 members declined to to carry out clinical examinations and genetic testing for their offspring. Therefore, long-term follow-up is needed in the future.

## Conclusion

The desmocollin2 was an important component of cardiac desmosome assembly, of which abnormality caused the dysfunction of cell–cell adhesions and intercellular gap junctions. The novel heterozygous *DSC2* p.K47Rfs*2 mutation remarkably and abnormally reduced the functional desmocollin2 expression, which may consequently induce the overlap phenotypes of LVNC and HCM, complicating AF, VT, and HF. The LVNC may be one of the important phenotypes for DSC2 pathogenic mutations.

## Data Availability

The data used in this study is not publicly available, but it might be available from the corresponding author upon reasonable request and permission from relevant Chinese Authorities.

## Abbreviations

CM: Cardiomyopathy
LVNC: Left ventricular noncompaction cardiomyopathy
HCM: Hypertrophic cardiomyopathy
DCM: Dilated cardiomyopathy
RCM: Restrictive cardiomyopathy
ACM: Arrhythmogenic cardiomyopathy
AF: Atrial fibrillation
VT: Ventricular tachycardia
HF: Heart failure
SCD: Sudden cardiac death
LVEF: Left ventricular ejection fraction
*MYH7*: Myosin heavy chain
*MYBPC3*: Protein-binding protein C myosin
*TPM1*: Tropomyosin alfa
*ACTC1*: Myocardial actin
*TNNT2*: Troponin T
*DTNA*: Alpha-distrobrevin
CMRI: Cardiac magnetic resonance imaging
ECGs: Electrocardiograms
NT-proBNP: The N-terminal of B-type natriuretic peptide precursor
LV: Left ventricle

## References

[1] Ross SB, Singer ES, Driscoll E (2020). Genetic architecture of left ventricular noncompaction in adults. Hum Genome Var.

[2] Arbustini E, Favalli V, Narula N, Serio A, Grasso M (2016). Left ventricular noncompaction: a distinct genetic cardiomyopathy. J Am Coll Cardiol.

[3] Ayesha B, Ahmed R, Gomceli U, Manrique C, Nicu M, Chilimuri S (2019). A case of isolated left ventricular non-compaction cardiomyopathy in a HIV patient presenting with acute heart failure. Cardiol Res.

[4] Sánchez Muñoz JJ, Esparza CM, Verdú PP, et al. Catheter ablation of ventricular arrhythmias in left ventricular noncompaction cardiomyopathy. Heart Rhythm. 2020. 10.1016/j.hrthm.2020.12.014 33346135

[5] Martinez A, Omar M, Kandah F, Ruiz J, Niazi A (2020). Left ventricular non-compaction presenting as cardio-embolic stroke. J Geriatr Cardiol.

[6] Aung N, Doimo S, Ricci F (2020). Prognostic significance of left ventricular noncompaction: systematic review and meta-analysis of observational studies. Circ Cardiovasc Imaging.

[7] Filho D, do Rêgo Aquino PL, de Souza Silva G, Fabro CB. Left ventricular noncompaction: new insights into a poorly understood disease. Curr Cardiol Rev. 2020. 10.2174/1573403X16666200716151015 PMC8226207 32674738

[8] Guo J, Li Z, Hao C (2019). A novel de novo CASZ1 heterozygous frameshift variant causes dilated cardiomyopathy and left ventricular noncompaction cardiomyopathy. Mol Genet Genomic Med.

[9] Vershinina T, Fomicheva Y, Muravyev A (2020). Genetic spectrum of left ventricular non-compaction in paediatric patients. Cardiology.

[10] Jenni R, Oechslin E, Schneider J, Attenhofer Jost C, Kaufmann PA (2001). Echocardiographic and pathoanatomical characteristics of isolated left ventricular non-compaction: a step towards classification as a distinct cardiomyopathy. Heart.

[11] Petersen SE, Selvanayagam JB, Wiesmann F (2005). Left ventricular non-compaction: insights from cardiovascular magnetic resonance imaging. J Am Coll Cardiol.

[12] Dong C, Wei P, Jian X (2015). Comparison and integration of deleteriousness prediction methods for nonsynonymous SNVs in whole exome sequencing studies. Hum Mol Genet.

[13] Li Q, Wang K (2017). InterVar: clinical interpretation of genetic variants by the 2015 ACMG-AMP guidelines. Am J Hum Genet.

[14] Richards S, Aziz N, Bale S (2015). Standards and guidelines for the interpretation of sequence variants: a joint consensus recommendation of the American College of Medical Genetics and Genomics and the Association for Molecular Pathology. Genet Med.

[15] Lin Y, Huang J, Zhao T, He S, Huang Z, Chen X, Fei H, Luo H, Liu H, Wu S, Lin X (2018). Compound and heterozygous mutations of DSG2 identified by Whole Exome Sequencing in arrhythmogenic right ventricular cardiomyopathy/dysplasia with ventricular tachycardia. J Electrocardiol.

[16] Lek M, Karczewski KJ, Minikel EV, Samocha KE, Banks E, Fennell T, Donnell-Luria AH, Ware JS, Hill AJ (2016). Analysis of protein-coding genetic variation in 60,706 humans. Nature.

[17] Kobayashi Y, Yang S, Nykamp K, Garcia J, Lincoln SE, Topper SE (2017). Pathogenic variant burden in the ExAC database: an empirical approach to evaluating population data for clinical variant interpretation. Genome Med.

[18] Abou Tayoun AN, Pesaran T, DiStefano MT, Oza A, Rehm HL, Biesecker LG, Harrison SM (2018). Recommendations for interpreting the loss of function PVS1 ACMG/AMP variant criterion. Hum Mutat.

[19] Sarquella-Brugada G, Fernandez-Falgueras A, Cesar S, Arbelo E, Coll M, Perez-Serra A, Puigmulé M, Iglesias A, Alcalde M, Vallverdú-Prats M, et al. Clinical impact of rare variants associated with inherited channelopathies: a 5-year update. Hum. Genet. 2021. 10.1007/s00439-021-02370-4 PMC9522753 34546463

[20] Campuzano O, Sarquella-Brugada G, Fernandez-Falgueras A, Coll M, Iglesias A, Ferrer-Costa C, Cesar S, Arbelo E, García-Álvarez A, Jordà P (2020). Reanalysis and reclassification of rare genetic variants associated with inherited arrhythmogenic syndromes. EBioMedicine.

[21] Musunuru K, Hershberger RE, Day SM, Klinedinst NJ, Landstrom AP, Parikh VN, Prakash S, Semsarian C, Sturm AC (2020). Genetic testing for inherited cardiovascular diseases: a scientific statement from the American Heart Association. Circ Genom Precis Med.

[22] Lahrouchi N, Raju H, Lodder EM, Papatheodorou E, Ware JS, Papadakis M, Tadros R, Cole D, Skinner JR, Crawford J (2017). Utility of post-mortem genetic testing in cases of sudden arrhythmic death syndrome. J Am Coll Cardiol.

[23] Costa S, Cerrone M, Saguner AM, Brunckhorst C, Delmar M, Duru F. Arrhythmogenic cardiomyopathy: An in-depth look at molecular mechanisms and clinical correlates. Trends Cardiovasc Med. 2020. 10.1016/j.tcm.2020.07.006 32738304

[24] Arunamata A, Stringer J, Balasubramanian S, Tacy TA, Silverman NH, Punn R (2019). Cardiac segmental strain analysis in pediatric left ventricular noncompaction cardiomyopathy. J Am Soc Echocardiogr.

[25] Towbin JA, Lorts A, Jefferies JL (2015). Left ventricular non-compaction cardiomyopathy. Lancet.

[26] Finsterer J, Stöllberger C, Towbin JA (2017). Left ventricular noncompaction cardiomyopathy: cardiac, neuromuscular, and genetic factors. Nat Rev Cardiol.

[27] Muser D, Liang JJ, Witschey WR (2017). Ventricular arrhythmias associated with left ventricular noncompaction: electrophysiologic characteristics, mapping, and ablation. Heart Rhythm.

[28] van Waning JI, Caliskan K, Hoedemaekers YM (2018). Genetics, clinical features, and long-term outcome of noncompaction cardiomyopathy. J Am Coll Cardiol.

[29] Abela M, D'Silva A (2018). Left ventricular trabeculations in athletes: epiphenomenon or phenotype of disease. Curr Treat Options Cardiovasc Med.

[30] Nozaki Y, Kato Y, Uike K (2020). Co-phenotype of left ventricular non-compaction cardiomyopathy and atypical catecholaminergic polymorphic ventricular tachycardia in association with R169Q, a ryanodine receptor type 2 missense mutation. Circ J.

[31] Precone V, Krasi G, Guerri G (2019). Cardiomyopathies. Acta Biomed.

[32] Richard P, Ader F, Roux M (2019). Targeted panel sequencing in adult patients with left ventricular non-compaction reveals a large genetic heterogeneity. Clin Genet.

[33] Sedaghat-Hamedani F, Haas J, Zhu F (2017). Clinical genetics and outcome of left ventricular non-compaction cardiomyopathy. Eur Heart J.

[34] Vergani V, Lazzeroni D, Peretto G (2020). Bridging the gap between hypertrabeculation phenotype, noncompaction phenotype and left ventricular noncompaction cardiomyopathy. J Cardiovasc Med (Hagerstown).

[35] Di Fusco SA, Lucà F, Madeo A (2020). Left ventricular noncompaction: diagnostic approach, prognostic evaluation, and management strategies. Cardiol Rev.

[36] Stämpfli SF, Gotschy A, Kiarostami P, et al. Right ventricular involvement in left ventricular non-compaction cardiomyopathy. Cardiol J. 2020. 10.5603/CJ.a2020.0095 PMC9170309 32648250

[37] Gurrieri F, Zollino M, Oliva A, Pascali V, Orteschi D, Pietrobono R, Camporeale A, Coll Vidal M, Partemi S, Brugada R (2013). Mild Beckwith–Wiedemann and severe long-QT syndrome due to deletion of the imprinting center 2 on chromosome 11p. Eur J Hum Genet.

[38] Chen H, Zhang W, Sun X (2013). Fkbp1a controls ventricular myocardium trabeculation and compaction by regulating endocardial Notch1 activity. Development.

[39] Shou W, Aghdasi B, Armstrong DL (1998). Cardiac defects and altered ryanodine receptor function in mice lacking FKBP12. Nature.

[40] Zhang W, Chen H, Qu X, Chang CP, Shou W (2013). Molecular mechanism of ventricular trabeculation/compaction and the pathogenesis of the left ventricular noncompaction cardiomyopathy (LVNC). Am J Med Genet C Semin Med Genet.

[41] Grego-Bessa J, Luna-Zurita L, del Monte G (2007). Notch signaling is essential for ventricular chamber development. Dev Cell.

[42] Sandireddy R, Cibi DM, Gupta P, et al. Semaphorin 3E/PlexinD1 signaling is required for cardiac ventricular compaction. JCI Insight. 2019;4(16). 10.1172/jci.insight.125908 PMC6777811 31434798

[43] Simons M, Mlodzik M (2008). Planar cell polarity signaling: from fly development to human disease. Annu Rev Genet.

[44] Wang Y, Nathans J (2007). Tissue/planar cell polarity in vertebrates: new insights and new questions. Development.

[45] Shimizu M, Ino H, Yamaguchi M, Terai H, Hayashi K, Kiyama M, Sakata K, Hayashi T, Inoue M, Kaneda T (2002). Chronologic electrocardiographic changes in patients with hypertrophic cardiomyopathy associated with cardiac troponin 1 mutation. Am Heart J.

[46] Vermij SH, Abriel H, van Veen TA (2017). Refining the molecular organization of the cardiac intercalated disc. Cardiovasc Res.

[47] Garrod D, Chidgey M (2008). Desmosome structure, composition and function. Biochim Biophys Acta.

[48] Getsios S, Amargo EV, Dusek RL (2004). Coordinated expression of desmoglein 1 and desmocollin 1 regulates intercellular adhesion. Differentiation.

[49] Kulikova O, Brodehl A, Kiseleva A, et al. The desmin (DES) mutation p.A337P is associated with left-ventricular non-compaction cardiomyopathy. Genes (Basel). 2021;12(1). 10.3390/genes12010121 PMC7835827 33478057

[50] Ramond F, Janin A, Di Filippo S (2017). Homozygous PKP2 deletion associated with neonatal left ventricle noncompaction. Clin Genet.

[51] Heuser A, Plovie ER, Ellinor PT (2006). Mutant desmocollin-2 causes arrhythmogenic right ventricular cardiomyopathy. Am J Hum Genet.

[52] Gerull B, Kirchner F, Chong JX, Tagoe J, Chandrasekharan K, Strohm O, Waggoner D, Ober C, Duff HJ (2013). Homozygous founder mutation in desmocollin-2 (DSC2) causes arrhythmogenic cardiomyopathy in the Hutterite population. Circ Cardiovasc Genet.

[53] Gehmlich K, Syrris P, Peskett E (2011). Mechanistic insights into arrhythmogenic right ventricular cardiomyopathy caused by desmocollin-2 mutations. Cardiovasc Res.

[54] Gehmlich K, Lambiase PD, Asimaki A (2011). A novel desmocollin-2 mutation reveals insights into the molecular link between desmosomes and gap junctions. Heart Rhythm.

[55] Kolegraff K, Nava P, Helms MN, Parkos CA, Nusrat A (2011). Loss of desmocollin-2 confers a tumorigenic phenotype to colonic epithelial cells through activation of Akt/β-catenin signaling. Mol Biol Cell.

[56] Semsarian C, Ingles J (2016). Molecular autopsy in victims of inherited arrhythmias. J Arrhythm.

[57] Dunn KE, Caleshu C, Cirino AL, Ho CY, Ashley EA (2013). A clinical approach to inherited hypertrophy: the use of family history in diagnosis, risk assessment, and management. Circ Cardiovasc Genet.

[58] Waldmüller S, Schroeder C, Sturm M (2015). Targeted 46-gene and clinical exome sequencing for mutations causing cardiomyopathies. Mol Cell Probes.

[59] Frustaci A, De Luca A, Guida V, et al. Novel α-actin gene mutation p.(Ala21Val) causing familial hypertrophic cardiomyopathy, myocardial noncompaction, and transmural crypts. Clinical-pathologic correlation. J Am Heart Assoc. 2018;7(4). 10.1161/JAHA.117.008068 PMC5850207 29440008

[60] Smith J, Owen T, Bhagwan JR (2018). Isogenic pairs of hiPSC-CMs with hypertrophic cardiomyopathy/LVNC-associated ACTC1 E99K mutation unveil differential functional deficits. Stem Cell Reports.

[61] Arad M, Penas-Lado M, Monserrat L (2005). Gene mutations in apical hypertrophic cardiomyopathy. Circulation.

[62] Klaassen S, Probst S, Oechslin E (2008). Mutations in sarcomere protein genes in left ventricular noncompaction. Circulation.

[63] Monserrat L, Barriales-Villa R, Hermida-Prieto M (2008). Apical hypertrophic cardiomyopathy and left ventricular non-compaction: two faces of the same disease. Heart.

[64] Monserrat L, Hermida-Prieto M, Fernandez X (2007). Mutation in the alpha-cardiac actin gene associated with apical hypertrophic cardiomyopathy, left ventricular non-compaction, and septal defects. Eur Heart J.

[65] Takasaki A, Hirono K, Hata Y (2018). Sarcomere gene variants act as a genetic trigger underlying the development of left ventricular noncompaction. Pediatr Res.

[66] Rodríguez-Serrano M, Domingo D, Igual B, Cano A, Medina P, Zorio E (2014). Familial left ventricular noncompaction associated with a novel mutation in the alpha-cardiac actin gene. Rev Esp Cardiol (Engl Ed).

[67] Sheikh N, Papadakis M, Wilson M (2018). Diagnostic yield of genetic testing in young athletes with T-wave inversion. Circulation.

[68] Tian T, Wang J, Wang H (2015). A low prevalence of sarcomeric gene variants in a Chinese cohort with left ventricular non-compaction. Heart Vessels.

[69] Girolami F, Iascone M, Tomberli B (2014). Novel α-actinin 2 variant associated with familial hypertrophic cardiomyopathy and juvenile atrial arrhythmias: a massively parallel sequencing study. Circ Cardiovasc Genet.

[70] Park J, Cho YG, Park HW, Cho JS (2021). Case report: novel likely pathogenic ACTN2 variant causing heterogeneous phenotype in a Korean family with left ventricular non-compaction. Front Pediatr.

[71] Al Senaidi K, Joshi N, Al-Nabhani M (2019). Phenotypic spectrum of ALPK3-related cardiomyopathy. Am J Med Genet A.

[72] Yilmaz S, Gokben S, Serdaroglu G (2016). The expanding phenotypic spectrum of ARFGEF2 gene mutation: cardiomyopathy and movement disorder. Brain Dev.

[73] Hirono K, Saito K, Munkhsaikhan U (2019). Familial left ventricular non-compaction is associated with a rare p..V407I variant in bone morphogenetic protein 10. Circ J.

[74] Chen H, Shi S, Acosta L (2004). BMP10 is essential for maintaining cardiac growth during murine cardiogenesis. Development.

[75] Cataldo S, Annoni GA, Marziliano N (2015). The perfect storm? Histiocytoid cardiomyopathy and compound CACNA2D1 and RANGRF mutation in a baby. Cardiol Young.

[76] Hoedemaekers YM, Caliskan K, Michels M (2010). The importance of genetic counseling, DNA diagnostics, and cardiologic family screening in left ventricular noncompaction cardiomyopathy. Circ Cardiovasc Genet.

[77] Darcha C, Laffargue F, Boutaud L, et al. Novel CDK10 variants with multicystic dysplastic kidney, left ventricular non-compaction, and a solitary median maxillary central incisor. Clin Genet. 2021. 10.1111/cge.13996 34114225

[78] Lan N, Fietz M, Pachter N, Paul V, Playford D (2018). A case of vascular Ehlers–Danlos Syndrome with a cardiomyopathy and multi-system involvement. Cardiovasc Pathol.

[79] Brodehl A, Gaertner-Rommel A, Milting H (2018). Molecular insights into cardiomyopathies associated with desmin (DES) mutations. Biophys Rev.

[80] Miszalski-Jamka K, Jefferies JL, Mazur W, et al. Novel genetic triggers and genotype-phenotype correlations in patients with left ventricular noncompaction. Circ Cardiovasc Genet. 2017;10(4). 10.1161/CIRCGENETICS.117.001763 PMC5665372 28798025

[81] Marakhonov AV, Brodehl A, Myasnikov RP (2019). Noncompaction cardiomyopathy is caused by a novel in-frame desmin (DES) deletion mutation within the 1A coiled-coil rod segment leading to a severe filament assembly defect. Hum Mutat.

[82] Tamiya R, Saito Y, Fukamachi D (2020). Desmin-related myopathy characterized by non-compaction cardiomyopathy, cardiac conduction defect, and coronary artery dissection. ESC Heart Fail.

[83] López-Ayala JM, Gómez-Milanés I, Sánchez Muñoz JJ (2014). Desmoplakin truncations and arrhythmogenic left ventricular cardiomyopathy: characterizing a phenotype. Europace.

[84] Williams T, Machann W, Kühler L (2011). Novel desmoplakin mutation: juvenile biventricular cardiomyopathy with left ventricular non-compaction and acantholytic palmoplantar keratoderma. Clin Res Cardiol.

[85] Cao Q, Shen Y, Liu X (2017). Phenotype and functional analyses in a transgenic mouse model of left ventricular noncompaction caused by a DTNA mutation. Int Heart J.

[86] Ichida F (2009). Left ventricular noncompaction. Circ J.

[87] Ichida F, Tsubata S, Bowles KR (2001). Novel gene mutations in patients with left ventricular noncompaction or Barth syndrome. Circulation.

[88] Xing Y, Ichida F, Matsuoka T (2006). Genetic analysis in patients with left ventricular noncompaction and evidence for genetic heterogeneity. Mol Genet Metab.

[89] Ishikawa T, Mishima H, Barc J (2020). Cardiac emerinopathy: a nonsyndromic nuclear envelopathy with increased risk of thromboembolic stroke due to progressive atrial standstill and left ventricular noncompaction. Circ Arrhythm Electrophysiol.

[90] Parent JJ, Towbin JA, Jefferies JL (2016). Fibrillin-1 gene mutations in left ventricular non-compaction cardiomyopathy. Pediatr Cardiol.

[91] Amiya E, Morita H, Hatano M (2016). Fukutin gene mutations that cause left ventricular noncompaction. Int J Cardiol.

[92] Ader F, De Groote P, Réant P (2019). FLNC pathogenic variants in patients with cardiomyopathies: prevalence and genotype-phenotype correlations. Clin Genet.

[93] Tang VT, Arscott P, Helms AS, Day SM (2018). Whole-exome sequencing reveals GATA4 and PTEN mutations as a potential digenic cause of left ventricular noncompaction. Circ Genom Precis Med.

[94] Ojala T, Nupponen I, Saloranta C (2015). Fetal left ventricular noncompaction cardiomyopathy and fatal outcome due to complete deficiency of mitochondrial trifunctional protein. Eur J Pediatr.

[95] Alonso-Fernández-Gatta M, Gallego-Delgado M, Caballero R, et al. A rare HCN4 variant with combined sinus bradycardia, left atrial dilatation, and hypertrabeculation/left ventricular noncompaction phenotype. Rev Esp Cardiol (Engl Ed). 2020. 10.1016/j.rec.2020.06.019 33008772

[96] Milano A, Vermeer AM, Lodder EM (2014). HCN4 mutations in multiple families with bradycardia and left ventricular noncompaction cardiomyopathy. J Am Coll Cardiol.

[97] Ishikawa T, Ohno S, Murakami T (2017). Sick sinus syndrome with HCN4 mutations shows early onset and frequent association with atrial fibrillation and left ventricular noncompaction. Heart Rhythm.

[98] Schweizer PA, Schröter J, Greiner S (2014). The symptom complex of familial sinus node dysfunction and myocardial noncompaction is associated with mutations in the HCN4 channel. J Am Coll Cardiol.

[99] Ponińska J, Michalak E, Śpiewak M, Lutyńska A, Płoski R, Bilińska ZT (2021). A novel HCN4 variant related to familial sinus bradycardia, left ventricular noncompaction, and thoracic aortic aneurysm. Pol Arch Intern Med.

[100] Yokoyama R, Kinoshita K, Hata Y (2018). A mutant HCN4 channel in a family with bradycardia, left bundle branch block, and left ventricular noncompaction. Heart Vessels.

[101] Mysliwiec MR, Bresnick EH, Lee Y (2011). Endothelial Jarid2/Jumonji is required for normal cardiac development and proper Notch1 expression. J Biol Chem.

[102] Xu B, Li K, Liu F (2021). Mexiletine shortened QT interval and reduced ventricular arrhythmias in a pedigree of type 2 long QT syndrome combined with left ventricular non-compaction. Int Heart J.

[103] Kharbanda M, Hunter A, Tennant S (2017). Long QT syndrome and left ventricular noncompaction in 4 family members across 2 generations with KCNQ1 mutation. Eur J Med Genet.

[104] Nakashima K, Kusakawa I, Yamamoto T (2013). A left ventricular noncompaction in a patient with long QT syndrome caused by a KCNQ1 mutation: a case report. Heart Vessels.

[105] Kazmirczak F, Martin CM, Shenoy C (2020). Left ventricular noncompaction and cardiogenic shock. Circulation.

[106] Codron P, Pautot V, Tassin A (2019). Abundant electrical myotonia and left ventricular noncompaction: unusual features of Danon disease due to a novel mutation in LAMP2 gene. Rev Neurol (Paris).

[107] Vatta M, Mohapatra B, Jimenez S (2003). Mutations in Cypher/ZASP in patients with dilated cardiomyopathy and left ventricular non-compaction. J Am Coll Cardiol.

[108] Xi Y, Ai T, De Lange E (2012). Loss of function of hNav1.5 by a ZASP1 mutation associated with intraventricular conduction disturbances in left ventricular noncompaction. Circ Arrhythm Electrophysiol.

[109] Li Z, Ai T, Samani K (2010). A ZASP missense mutation, S196L, leads to cytoskeletal and electrical abnormalities in a mouse model of cardiomyopathy. Circ Arrhythm Electrophysiol.

[110] Pignatelli RH, McMahon CJ, Dreyer WJ (2003). Clinical characterization of left ventricular noncompaction in children: a relatively common form of cardiomyopathy. Circulation.

[111] Theis JL, Bos JM, Bartleson VB (2006). Echocardiographic-determined septal morphology in Z-disc hypertrophic cardiomyopathy. Biochem Biophys Res Commun.

[112] Liu Z, Shan H, Huang J, Li N, Hou C, Pu J (2016). A novel lamin A/C gene missense mutation (445 V > E) in immunoglobulin-like fold associated with left ventricular non-compaction. Europace.

[113] Parent JJ, Towbin JA, Jefferies JL (2015). Left ventricular noncompaction in a family with lamin A/C gene mutation. Tex Heart Inst J.

[114] Rankin J, Auer-Grumbach M, Bagg W (2008). Extreme phenotypic diversity and nonpenetrance in families with the LMNA gene mutation R644C. Am J Med Genet A.

[115] Luxán G, Casanova JC, Martínez-Poveda B (2013). Mutations in the NOTCH pathway regulator MIB1 cause left ventricular noncompaction cardiomyopathy. Nat Med.

[116] Piccolo P, Attanasio S, Secco I (2017). MIB2 variants altering NOTCH signalling result in left ventricle hypertrabeculation/non-compaction and are associated with Ménétrier-like gastropathy. Hum Mol Genet.

[117] Eldomery MK, Akdemir ZC, Vögtle FN (2016). MIPEP recessive variants cause a syndrome of left ventricular non-compaction, hypotonia, and infantile death. Genome Med.

[118] Tanpaiboon P, Sloan JL, Callahan PF (2013). Noncompaction of the ventricular myocardium and hydrops fetalis in cobalamin C disease. JIMD Rep.

[119] Probst S, Oechslin E, Schuler P (2011). Sarcomere gene mutations in isolated left ventricular noncompaction cardiomyopathy do not predict clinical phenotype. Circ Cardiovasc Genet.

[120] Camuglia AC, Younger JF, McGaughran J, Lo A, Atherton JJ (2013). Cardiac myosin-binding protein C gene mutation expressed as hypertrophic cardiomyopathy and left ventricular noncompaction within two families: insights from cardiac magnetic resonance in clinical screening: Camuglia MYBPC3 gene mutation and MRI. Int J Cardiol.

[121] Liu S, Xie Y, Zhang H (2020). Multiple genetic variants in adolescent patients with left ventricular noncompaction cardiomyopathy. Int J Cardiol.

[122] Wessels MW, Herkert JC, Frohn-Mulder IM (2015). Compound heterozygous or homozygous truncating MYBPC3 mutations cause lethal cardiomyopathy with features of noncompaction and septal defects. Eur J Hum Genet.

[123] Ripoll Vera T, Monserrat Iglesias L, Hermida Prieto M (2010). The R820W mutation in the MYBPC3 gene, associated with hypertrophic cardiomyopathy in cats, causes hypertrophic cardiomyopathy and left ventricular non-compaction in humans. Int J Cardiol.

[124] Kolokotronis K, Kühnisch J, Klopocki E (2019). Biallelic mutation in MYH7 and MYBPC3 leads to severe cardiomyopathy with left ventricular noncompaction phenotype. Hum Mutat.

[125] Schaefer E, Helms P, Marcellin L (2014). Next-generation sequencing (NGS) as a fast molecular diagnosis tool for left ventricular noncompaction in an infant with compound mutations in the MYBPC3 gene. Eur J Med Genet.

[126] Haberer K, Buffo-Sequeira I, Chudley AE, Spriggs E, Sergi C (2014). A case of an infant with compound heterozygous mutations for hypertrophic cardiomyopathy producing a phenotype of left ventricular noncompaction. Can J Cardiol.

[127] Dellefave LM, Pytel P, Mewborn S (2009). Sarcomere mutations in cardiomyopathy with left ventricular hypertrabeculation. Circ Cardiovasc Genet.

[128] Prada CE, Jefferies JL, Grenier MA (2012). Malonyl coenzyme A decarboxylase deficiency: early dietary restriction and time course of cardiomyopathy. Pediatrics.

[129] Bainbridge MN, Davis EE, Choi WY (2015). Loss of function mutations in NNT are associated with left ventricular noncompaction. Circ Cardiovasc Genet.

[130] Vermeer AM, van Engelen K, Postma AV (2013). Ebstein anomaly associated with left ventricular noncompaction: an autosomal dominant condition that can be caused by mutations in MYH7. Am J Med Genet C Semin Med Genet.

[131] Postma AV, van Engelen K, van de Meerakker J (2011). Mutations in the sarcomere gene MYH7 in Ebstein anomaly. Circ Cardiovasc Genet.

[132] Hirono K, Hata Y, Ibuki K, Yoshimura N (2014). Familial Ebstein's anomaly, left ventricular noncompaction, and ventricular septal defect associated with an MYH7 mutation. J Thorac Cardiovasc Surg.

[133] Latus H, Yerebakan C, Schranz D, Akintuerk H (2014). Right ventricular failure from severe pulmonary hypertension after surgery for shone complex: back to fetal physiology with reducting, atrioseptectomy, and bilateral pulmonary arterial banding. J Thorac Cardiovasc Surg.

[134] Yang J, Zhu M, Wang Y (2015). Whole-exome sequencing identify a new mutation of MYH7 in a Chinese family with left ventricular noncompaction. Gene.

[135] Aksel T, Choe YuE, Sutton S, Ruppel KM, Spudich JA (2015). Ensemble force changes that result from human cardiac myosin mutations and a small-molecule effector. Cell Rep.

[136] Kaneda T, Naruse C, Kawashima A (2008). A novel beta-myosin heavy chain gene mutation, p.Met531Arg, identified in isolated left ventricular non-compaction in humans, results in left ventricular hypertrophy that progresses to dilation in a mouse model. Clin Sci (Lond).

[137] Uchiyama T, Yoshimura K, Kaneko K (2012). Surgical repair of left ventricular noncompaction in a patient with a novel mutation of the myosin heavy chain 7 gene. Tohoku J Exp Med.

[138] Sunbul M, Ozben B, Mutlu B (2013). Two different cardiomyopathies in a single patient : hypertrophic cardiomyopathy and left ventricular noncompaction. Herz.

[139] Esposito T, Sampaolo S, Limongelli G (2013). Digenic mutational inheritance of the integrin alpha 7 and the myosin heavy chain 7B genes causes congenital myopathy with left ventricular non-compact cardiomyopathy. Orphanet J Rare Dis.

[140] Hoedemaekers YM, Cohen-Overbeek TE, Frohn-Mulder IM, Dooijes D, Majoor-Krakauer DF (2013). Prenatal ultrasound diagnosis of MYH7 non-compaction cardiomyopathy. Ultrasound Obstet Gynecol.

[141] Ruggiero L, Fiorillo C, Gibertini S (2015). A rare mutation in MYH7 gene occurs with overlapping phenotype. Biochem Biophys Res Commun.

[142] Finsterer J, Brandau O, Stöllberger C, Wallefeld W, Laing NG, Laccone F (2014). Distal myosin heavy chain-7 myopathy due to the novel transition c.5566G>A (p.E1856K) with high interfamilial cardiac variability and putative anticipation. Neuromuscul Disord.

[143] Finsterer J, Stöllberger C, Hasun M, Riedhammer K, Wagner M (2020). Multisystem myotilinopathy, including myopathy and left ventricular noncompaction, due to the MYOT variant c.179C>T. Case Rep Cardiol.

[144] Perrot A, Tomasov P, Villard E (2016). Mutations in NEBL encoding the cardiac Z-disk protein nebulette are associated with various cardiomyopathies. Arch Med Sci.

[145] Palomino Doza J, Salguero-Bodes R, de la Parte M, Arribas-Ynsaurriaga F (2018). Association between mutations in the NKX2.5 homeobox, atrial septal defects, ventricular noncompaction and sudden cardiac death. Rev Esp Cardiol (Engl Ed).

[146] Ouyang P, Saarel E, Bai Y (2011). A de novo mutation in NKX2.5 associated with atrial septal defects, ventricular noncompaction, syncope and sudden death. Clin Chim Acta.

[147] Sun H, Han L, Zhang X (2020). Case report: characterization of a novel NONO intronic mutation in a fetus With X-linked syndromic mental retardation-34. Front Genet.

[148] Reinstein E, Tzur S, Cohen R, Bormans C, Behar DM (2016). Intellectual disability and non-compaction cardiomyopathy with a de novo NONO mutation identified by exome sequencing. Eur J Hum Genet.

[149] Gannon T, Perveen R, Schlecht H (2015). Further delineation of the KAT6B molecular and phenotypic spectrum. Eur J Hum Genet.

[150] Sewani M, Nugent K, Blackburn PR (2020). Further delineation of the phenotypic spectrum associated with hemizygous loss-of-function variants in NONO. Am J Med Genet A.

[151] Scott DA, Hernandez-Garcia A, Azamian MS (2017). Congenital heart defects and left ventricular non-compaction in males with loss-of-function variants in NONO. J Med Genet.

[152] Carlston CM, Bleyl SB, Andrews A (2019). Expanding the genetic and clinical spectrum of the NONO-associated X-linked intellectual disability syndrome. Am J Med Genet A.

[153] Sun H, Zhou X, Hao X, Zhang Y, Zhang H, He Y (2020). Characteristics of cardiac phenotype in prenatal familial cases with NONO mutations. Circ Genom Precis Med.

[154] Yang J, Bücker S, Jungblut B (2012). Inhibition of Notch2 by Numb/Numblike controls myocardial compaction in the heart. Cardiovasc Res.

[155] Rowland TJ, Graw SL, Sweet ME, Gigli M, Taylor MR, Mestroni L (2016). Obscurin variants in patients with left ventricular noncompaction. J Am Coll Cardiol.

[156] Muhammad E, Levitas A, Singh SR (2015). PLEKHM2 mutation leads to abnormal localization of lysosomes, impaired autophagy flux and associates with recessive dilated cardiomyopathy and left ventricular noncompaction. Hum Mol Genet.

[157] Long PA, Evans JM, Olson TM (2017). Diagnostic yield of whole exome sequencing in pediatric dilated cardiomyopathy. J Cardiovasc Dev Dis.

[158] Delplancq G, Tarris G, Vitobello A (2020). Cardiomyopathy due to PRDM16 mutation: first description of a fetal presentation, with possible modifier genes. Am J Med Genet C Semin Med Genet.

[159] Kim K, Kang MG, Park HW (2018). A rare case of left ventricular noncompaction in LEOPARD syndrome. J Cardiovasc Ultrasound.

[160] Sun Q, Guo J, Hao C (2020). Whole-exome sequencing reveals two de novo variants in the RBM20 gene in two Chinese patients with left ventricular non-compaction cardiomyopathy. Pediatr Investig.

[161] Leung C, Engineer A, Kim MY, Lu X, Feng Q (2021). Myocardium-specific deletion of Rac1 causes ventricular noncompaction and outflow tract defects. J Cardiovasc Dev Dis..

[162] Martinez HR, Niu MC, Sutton VR, Pignatelli R, Vatta M, Jefferies JL (2011). Coffin–Lowry syndrome and left ventricular noncompaction cardiomyopathy with a restrictive pattern. Am J Med Genet A.

[163] Bhuiyan ZA, van den Berg MP, van Tintelen JP (2007). Expanding spectrum of human RYR2-related disease: new electrocardiographic, structural, and genetic features. Circulation.

[164] Campbell MJ, Czosek RJ, Hinton RB, Miller EM (2015). Exon 3 deletion of ryanodine receptor causes left ventricular noncompaction, worsening catecholaminergic polymorphic ventricular tachycardia, and sudden cardiac arrest. Am J Med Genet A.

[165] Ohno S, Omura M, Kawamura M (2014). Exon 3 deletion of RYR2 encoding cardiac ryanodine receptor is associated with left ventricular non-compaction. Europace.

[166] Roston TM, Guo W, Krahn AD (2017). A novel RYR2 loss-of-function mutation (I4855M) is associated with left ventricular non-compaction and atypical catecholaminergic polymorphic ventricular tachycardia. J Electrocardiol.

[167] Shan L, Makita N, Xing Y (2008). SCN5A variants in Japanese patients with left ventricular noncompaction and arrhythmia. Mol Genet Metab.

[168] Alston CL, Ceccatelli Berti C, Blakely EL (2015). A recessive homozygous p.Asp92Gly SDHD mutation causes prenatal cardiomyopathy and a severe mitochondrial complex II deficiency. Hum Genet.

[169] Li L, Huang L, Lin S, Luo Y, Fang Q (2017). Discordant phenotypes in monozygotic twins with 16p11.2 microdeletions including the SH2B1 gene. Am J Med Genet A.

[170] Lin W, Li D, Cheng L (2018). Zinc transporter Slc39a8 is essential for cardiac ventricular compaction. J Clin Invest.

[171] Wenger TL, Chow P, Randle SC (2017). Novel findings of left ventricular non-compaction cardiomyopathy, microform cleft lip and poor vision in patient with SMC1A-associated Cornelia de Lange syndrome. Am J Med Genet A.

[172] Sun H, Yu S, Zhou X, Han L, Zhang H, He Y (2020). Expanding the phenotype of STRA6-related disorder to include left ventricular non-compaction. Mol Genet Genomic Med.

[173] Brady AN, Shehata BM, Fernhoff PM (2006). X-linked fetal cardiomyopathy caused by a novel mutation in the TAZ gene. Prenat Diagn.

[174] Chang B, Momoi N, Shan L (2010). Gonadal mosaicism of a TAZ (G4.5) mutation in a Japanese family with Barth syndrome and left ventricular noncompaction. Mol Genet Metab.

[175] Momoi N, Chang B, Takeda I, Aoyagi Y, Endo K, Ichida F (2012). Differing clinical courses and outcomes in two siblings with Barth syndrome and left ventricular noncompaction. Eur J Pediatr.

[176] Kenton AB, Sanchez X, Coveler KJ (2004). Isolated left ventricular noncompaction is rarely caused by mutations in G4.5, alpha-dystrobrevin and FK Binding Protein-12. Mol Genet Metab.

[177] Wang J, Guo Y, Huang M (2017). Identification of TAZ mutations in pediatric patients with cardiomyopathy by targeted next-generation sequencing in a Chinese cohort. Orphanet J Rare Dis.

[178] Chen R, Tsuji T, Ichida F (2002). Mutation analysis of the G4.5 gene in patients with isolated left ventricular noncompaction. Mol Genet Metab.

[179] Ronvelia D, Greenwood J, Platt J, Hakim S, Zaragoza MV (2012). Intrafamilial variability for novel TAZ gene mutation: Barth syndrome with dilated cardiomyopathy and heart failure in an infant and left ventricular noncompaction in his great-uncle. Mol Genet Metab.

[180] Karkucinska-Wieckowska A, Trubicka J, Werner B (2013). Left ventricular noncompaction (LVNC) and low mitochondrial membrane potential are specific for Barth syndrome. J Inherit Metab Dis.

[181] Ross SB, Bagnall RD, Yeates L, Sy RW, Semsarian C (2018). Holt-Oram syndrome in two families diagnosed with left ventricular noncompaction and conduction disease. Heart Rhythm Case Rep.

[182] Miyao N, Hata Y, Izumi H (2020). TBX5 R264K acts as a modifier to develop dilated cardiomyopathy in mice independently of T-box pathway. PLoS ONE.

[183] Kodo K, Ong SG, Jahanbani F (2016). iPSC-derived cardiomyocytes reveal abnormal TGF-β signalling in left ventricular non-compaction cardiomyopathy. Nat Cell Biol.

[184] Hirono K, Ichida F, Nishio N (2019). Mitochondrial complex deficiency by novel compound heterozygous TMEM70 variants and correlation with developmental delay, undescended testicle, and left ventricular noncompaction in a Japanese patient: a case report. Clin Case Rep.

[185] Fujino M, Tsuda E, Hirono K (2018). The TNNI3 Arg192His mutation in a 13-year-old girl with left ventricular noncompaction. J Cardiol Cases.

[186] Luedde M, Ehlermann P, Weichenhan D (2010). Severe familial left ventricular non-compaction cardiomyopathy due to a novel troponin T (TNNT2) mutation. Cardiovasc Res.

[187] Mogensen J, Murphy RT, Shaw T (2004). Severe disease expression of cardiac troponin C and T mutations in patients with idiopathic dilated cardiomyopathy. J Am Coll Cardiol.

[188] Chang B, Nishizawa T, Furutani M (2011). Identification of a novel TPM1 mutation in a family with left ventricular noncompaction and sudden death. Mol Genet Metab.

[189] Nijak A, Alaerts M, Kuiperi C (2018). Left ventricular non-compaction with Ebstein anomaly attributed to a TPM1 mutation. Eur J Med Genet.

[190] Kelle AM, Bentley SJ, Rohena LO, Cabalka AK, Olson TM (2016). Ebstein anomaly, left ventricular non-compaction, and early onset heart failure associated with a de novo α-tropomyosin gene mutation. Am J Med Genet A.

[191] Hastings R, de Villiers CP, Hooper C (2016). Combination of whole genome sequencing, linkage, and functional studies implicates a missense mutation in titin as a cause of autosomal dominant cardiomyopathy with features of left ventricular noncompaction. Circ Cardiovasc Genet.

[192] Chauveau C, Bonnemann CG, Julien C (2014). Recessive TTN truncating mutations define novel forms of core myopathy with heart disease. Hum Mol Genet.

[193] Chang B, Gorbea C, Lezin G (2013). 14-3-3ε gene variants in a Japanese patient with left ventricular noncompaction and hypoplasia of the corpus callosum. Gene.

[194] Tang S, Batra A, Zhang Y, Ebenroth ES, Huang T (2010). Left ventricular noncompaction is associated with mutations in the mitochondrial genome. Mitochondrion.

[195] Zarrouk Mahjoub S, Mehri S, Ourda F (2011). Transition m.3308T>C in the ND1 gene is associated with left ventricular hypertrabeculation/noncompaction. Cardiology.

[196] Zarrouk Mahjoub S, Mehri S, Ourda F (2012). Pathogenicity of the transition m.3308T>C in left ventricular hypertrabeculation/noncompaction. Cardiology.

